# The biosynthesis of EGCG, theanine and caffeine in response to temperature is mediated by hormone signal transduction factors in tea plant (*Camellia sinensis* L.)

**DOI:** 10.3389/fpls.2023.1149182

**Published:** 2023-03-23

**Authors:** Qiufang Zhu, Lijia Liu, Xiaofeng Lu, Xinxin Du, Ping Xiang, Bosi Cheng, Meng Tan, Jiaxin Huang, Lijiao Wu, Weilong Kong, Yutao Shi, Liangyu Wu, Jinke Lin

**Affiliations:** ^1^ College of Horticulture, Fujian Agriculture and Forestry University, Fuzhou, China; ^2^ College of Life and Environmental Science, Hunan University of Arts and Science, Changde, China; ^3^ Institute of Photobiological Industry, Fujian Sanan Sino-Science Photobiotech Co., Ltd, Xiamen, China; ^4^ Shenzhen Branch, Guangdong Laboratory for Lingnan Modern Agriculture, Genome Analysis Laboratory of the Ministry of Agriculture, Agricultural Genomics Institute at Shenzhen, Chinese Academy of Agricultural Sciences, Shenzhen, China; ^5^ College of Tea and Food Sciences, Wuyi University, Wuyishan, China

**Keywords:** *Camellia sinensis* (L.) O. Kuntze, temperature, endogenous hormone, plant hormone signal transduction, EGCG, theanine, caffeine, biosynthesis

## Abstract

As the main flavor components of tea, the contents of epigallocatechin-3-gallate (EGCG), theanine and caffeine are regulated by ambient temperature. However, whether the biosynthesis of EGCG, theanine and caffeine in response to temperature is regulated by endogenous hormones and its mechanism is still unclear. In this study, tea cuttings cultivated in the phytotron which treated at different temperatures 15℃, 20℃, 25℃ and 30℃, respectively. The UPLC and ESI-HPLC-MS/MS were used to determine the contents of EGCG, theanine, caffeine and the contents of phytohormones in one leaf and a bud. The results showed that indoleacetic acid (IAA), gibberellin 1(GA1) and gibberellin 3 (GA3) were significantly correlated with the content of EGCG; Jasmonic acid (JA), jasmonate-isoleucine (JA-Ile) and methyl jasmonate (MeJA) were strongly correlated with theanine content; IAA, GA1 and gibberellin 4 (GA4) were significantly correlated with caffeine content at different temperatures. In order to explore the internal intricate relationships between the biosynthesis of these three main taste components, endogenous hormones, and structural genes in tea plants, we used multi-omics and multidimensional correlation analysis to speculate the regulatory mechanisms: IAA, GA1 and GA3 up-regulated the expressions of *chalcone synthase* (*CsCHS*) and *trans-cinnamate 4-monooxygenase* (*CsC4H*) mediated by the signal transduction factors *auxin-responsive protein IAA* (*CsIAA*) and *DELLA protein* (*CsDELLA*), respectively, which promoted the biosynthesis of EGCG; IAA, GA3 and GA1 up-regulated the expression of *CsCHS* and *anthocyanidin synthase* (*CsANS*) mediated by *CsIAA* and *CsDELLA*, respectively, via the transcription factor *WRKY DNA-binding protein* (*CsWRKY*), and promoted the biosynthesis of EGCG; JA, JA-Ile and MeJA jointly up-regulated the expression of *carbonic anhydrase* (*CsCA*) and down-regulated the expression of *glutamate decarboxylase* (*CsgadB*) mediated by the signal transduction factors *jasmonate ZIM domain-containing protein* (*CsJAZ*), and promoted the biosynthesis of theanine; JA, JA-Ile and MeJA also jointly inhibited the expression of *CsgadB* mediated by *CsJAZ* via the transcription factor *CsWRKY* and *AP2 family protein* (*CsAP2*), which promoted the biosynthesis of theanine; IAA inhibited the expression of *adenylosuccinate synthase* (*CspurA*) mediated by *CsIAA* via the transcription factor *CsWRKY*; GA1 and gibberellin 4 (GA4) inhibited the expression of *CspurA* mediated by *CsDELLA* through the transcription factor *CsWRKY*, which promoted the biosynthesis of caffeine. In conclusion, we revealed the underlying mechanism of the biosynthesis of the main taste components in tea plant in response to temperature was mediated by hormone signal transduction factors, which provided novel insights into improving the quality of tea.

## 1 Introduction

Tea is one of the most popular non-alcoholic beverages due to the unique flavor and multi healthy benefits given by the natural products such as EGCG, theanine and caffeine. EGCG is a main flavor component in tea (Zhao et al., 2020), and involved in anti-cancer (Zhang et al., 2020a; Han et al., 2021), anti-hypertensive (Luo et al., 2020; Del et al., 2022), anti-diabetics effects (Lin and Lin, 2008; Zhu et al., 2022). Theanine has caramel and sweet flavors (Narukawa et al., 2008), and can relax nerves, improve attention and cognition (Giesbrecht et al., 2013; Tamano et al., 2013; Kahathuduwa et al., 2017), prevent acute liver toxicity induced by adriamycin and liver steatosis caused by non-alcohol (Nagai et al., 2015; Liang et al., 2022). Caffeine has bitter taste (Lipchock et al., 2017) and can refresh and resist fatigue (Xu et al., 2007; Zeng et al., 2022), diuresis, prevent retinopathy (Zhang et al., 2017), improve memory disorders (Cunha and Agostinho, 2010).

During the growth and development, there are many factors that affecting the biosynthesis of EGCG, theanine and caffeine in tea plant, including meteorological factors (such as light (Xiang et al., 2021b), temperature (Xiang et al., 2021a; Xiang et al., 2023) and humidity), endogenous hormones (Fang et al., 2022) and so on. As an important environmental factor, temperature directly influences the contents of EGCG, theanine and caffeine in tea plant. Previous studies showed that the contents of epigallocatechin (EGC), epicatechin (EC), epicatechin gallate (ECG) and epigallocatechin gallate (EGCG) in tea were increased along with the elevation of daily temperature (Wang et al., 2011). The expression of *flavanone 4-reductase* (*DFR*) and *anthocyanidin reductase* (*ANR*) genes were up-regulated in tea plants that cultivated in the greenhouse, which promoted the accumulation of catechins and EGCG (Liu et al., 2015a). The EGCG content is positively relative to the temperature under artificial temperature control (Xiang et al., 2021a). The theanine content in tea plant was reduced at high temperature (Li et al., 2018). As for caffeine, it’s content was increased at high temperature (Li et al., 2020).

As a class of low-molecular-weight compounds, endogenous hormones also affect the biosynthesis of the taste components in tea plant. Zhao had found that IAA, zeatin (ZA), abscisic acid (ABA), and JA were positively related to the accumulation of gallated catechin, caffeine, and theanine, and SA was negatively correlated with these compounds in different leaf positions of tea plants (Zhao et al., 2020). Exogenous melatonin could improve the adverse effects of moderate high temperatures on tea quality by increasing the epigallocatechin-3-gallate and theanine biosynthesis in tea plant (Li et al., 2020). The exogenous JA and SA could regulate the content of catechins, SA affected the content of caffeine, and ABA affected the content of theaflavin (Liao et al., 2019). Exogenous application of BR could increase the content of theanine, tea polyphenols and free amino acids under sub-high temperature, and improve the quality of summer tea (Li et al., 2018). Sunlight withering may regulate the formation of flavor substances by reducing IAA and GA content (Zhu et al., 2020).

The biosynthesis of taste components in tea plant is not only affected by temperature, but also regulated by plant hormones. However, the internal intricate relationships between the biosynthesis of EGCG, theanine and caffeine and endogenous hormones in response to temperature in tea plant and its underlying mechanism are still unclear. In this study, tea plants were cultivated in artificial climate chamber to control the ambient temperature, the study on the internal relationships between the biosynthesis of EGCG, theanine and caffeine and endogenous hormones, signal transduction factors, transcription factors and structural genes in tea plant at different temperature treatments were explored, in order to provide a new insight into understanding the regulation of fresh leaf quality of tea plant.

## 2 Materials and methods

### 2.1 Plant materials and temperature treatment

One-year-old cuttings of *Camellia sinensis* cv. ‘Huangdan’ were purchased from Qianhe Tea Cooperative of Anxi County. The tea cuttings were transplanted into a seedling bag with a diameter of 16 cm and a height of 18 cm, and then moved into an artificial climate chamber located in Fujian Sanan Sino-Science Photobiotech Co., Ltd (Anxi County, Quanzhou City, Fujian, China, 193.7 m, 118°1′30″E, 25°13′19″N). Different temperature treatments were started in January 2021. Four different temperature treatments were set: 15°C, 20°C, 25°C, 30°C. Each treatment contained 100 tea cuttings. After 90 days, each 30 tea cuttings were sampled for one biological repetition, and three biological repetitions were performed. The parameters of other environmental conditions in the artificial climate chamber were set as follows: light intensity was 200 µmol·m^-2^·s^-1^, air humidity was 70%, CO_2_ concentration was 750 µmol·mol^-1^, which were referred to Xiang (Xiang et al., 2021a; Xiang et al., 2023), and the nutrient solution was optimized according to the formula of Shigeki et al. (1985). The tea shoots composed of one leaf and a bud were picked, some were dried in an oven in two stages (120°C 10min, 90°C 30min) and stored at -20°C for the determination of EGCG, theanine and caffeine contents, and the others were quickly frozen in liquid nitrogen, then transferred to a refrigerator at - 80°C for further analysis. All the samples were composed of three biological replicates.

### 2.2 Determination of EGCG, caffeine and theanine content

The contents of EGCG and caffeine were measured using UPLC system equipped with Waters Acquity UPLC HSS T3 column (2.1×100 mm, 1.8 μm). The detection method of EGCG and caffeine content was referred to Lin et al. (2017). The extraction of theanine was as follow: of the freeze-dried tea leaves was ground with a mortar in liquid nitrogen. The tea powder (150 mg) was dissolved with 5 mL deionized boiling water, then incubated in a water bath at 100°C for 20min (Wang et al., 2012). After centrifugation at 4,000 rpm for 10 min, the residues were re-extracted once as described above. The supernatants were combined and diluted with water to a volume of 10 mL. Then the supernatant was filtered through a 0.45 μm membrane (Tai et al., 2015). The UPLC system Waters ACCQ-TAG ULTRAC18 (4.6×100 mm, 2.5 μm) was used to measure the content of theanine. The mobile phase consisted of 20 mmol/L ammonium formate aqueous solution (A), 50% acetonitrile (B) with the flow rate of 0.7mL/min. The column oven temperature was set to 55°C. The detection wavelength was set to 260 nm for analysis.

### 2.3 HPLC-MS analysis of endogenous hormone content

Using ESI-HPLC-MS/MS methods to measure the content of IAA, 3-Indole butyric acid (IBA), Indole acetyl aspartic acid (IAA-Asp), GA1, GA3, GA4, zeatin, trans-zeatin-riboside (tZR), isopentenyladenosine (iPR), isoamyl alkenyl adenine (iP), ABA, JA, JA-Ile, MeJA, salicylic acid (SA), Methyl salicylate (MeSA). About 200 mg of plant tissue was ground into a fine powder in liquid nitrogen and then extracted twice with acetonitrile and purified with a Poroshell 120 SB-C18 column. The quantification of hormone was determined by LC-MS (Agilent 1290 Infinity-SCIEX 6500Qtrap, https://www.agilent.com/). These experiments were repeated three times with similar results (Pan et al., 2010; Liu et al., 2014; Cui et al., 2021). This work was done by RUIYUAN Biotechnology Co., Ltd (www.bestofbest.top).

### 2.4 RNA extraction, RNA-seq, and bioinformatics analysis

The total RNA of the tea samples was extracted with CTAB lysate containing 2% mercaptoethanol. The RNA extraction, RNA-sequencing, and bioinformatics analysis were performed in a commercial company (BGI Genomics, Shenzhen, China). The sequencing platform was DNBSEQ. The reads with low quality, joint contamination and high content of unknown base N were filtered out from raw data to obtain clean reads, then the clean reads were mapped according to reference tea genome of cv. ‘Huangdan’ genome (https://bigd.big.ac.cn/, general number PRJCA003382) (**Supplementary Table 1**). Using the Trinity built-in script “align_and_estimate_abundation.pl” to blast and quantify. Blast software was set to bowtie, the quantitative method was set as RSEM and differential expression analysis was performed by edgeR.

### 2.5 Validation of high-throughput sequencing technologies RNA-Seq by qRT-PCR

Integrating the results of significant correlation, transcription factor binding site prediction and the promoter cis-element prediction, we obtain the 36 hub genes. To verify the accuracy of the transcriptome data, 36 hub genes were selected for expression level validation. Primers were designed using Primer3Plus (https://www.bioinformatics.nl/cgi-bin/primer3plus/primer3plus.cgi/), and the primer sequences are shown in **Supplementary Table 2**. Quantitative real-time PCR (qRT-PCR) was performed on ABI 7500 HT Real-time system (ABI Company, USA) using SuperReal PreMix Plus (SYBP Green) (FP205-02, Tiangen Company, China) in January 2023. Three biological replicates were analyzed. Using the *beta-actin* (*Csβ-Actin*) gene as an internal reference gene, the relative expression was quantified by the 2^−ΔΔCt^ method.

### 2.6 Data analysis

Each sample was composed of three biological replicates. The T-test and correlation analysis were performed on SPSS 24.0. With 16 plant hormones as X variable and EGCG, theanine and caffeine as Y variable, the orthogonal projection to latent structures-discriminant analysis (OPLS) was conducted on SIMCA 14.1. Heatmap was produced on TBtools (Chen et al., 2020). Gene ontology (GO) and Kyoto Encyclopedia of Genes and Genomes (KEGG) enrichment analysis of differentially expressed genes (DEGs) were conducted by OmicShare platform (https://www.omicshare.com/tools/). The top 20 pathways were screened by *P*<0.05 to draw the GO and KEGG bubble maps.

## 3 Results

### 3.1 The content variation of EGCG, theanine and caffeine in response to temperature

**Figure 1:**
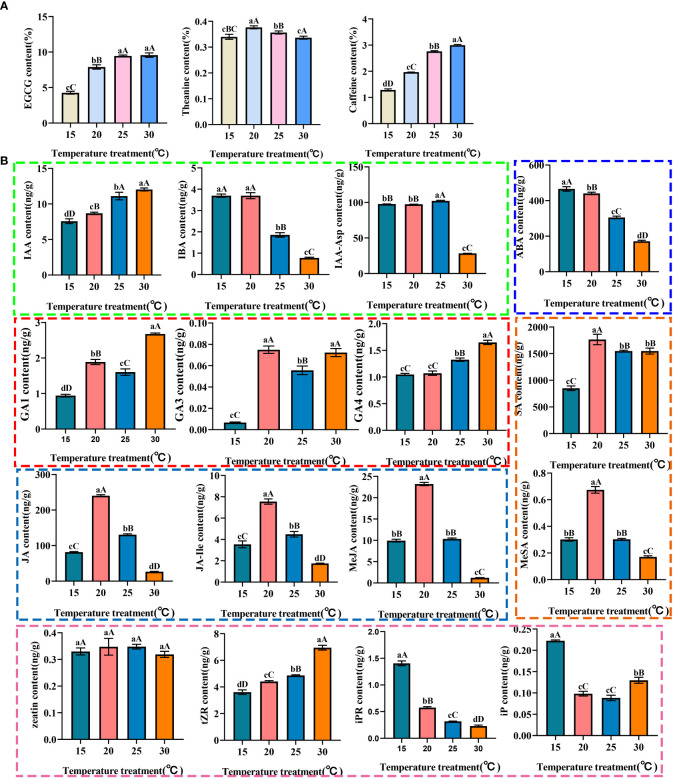
The contents of EGCG, theanine, caffeine **(A)** and endogenous hormones **(B)** in tea plant at different temperatures. Different lowercase letters represent *P*<0.05, which is significant between pairwise; Different capital letters represent *P*<0.01, which is extremely significant between pairwise.

As the main taste components in tea, the contents of EGCG, theanine and caffeine were regulated by temperatures (**Figure 1A**; **Supplementary Table 3**). Compared with 15°C, the content of EGCG and caffeine in tea plant showed significant differences in the groups cultivated at 20°C, 25°C and 30°C. Moreover, the EGCG and caffeine contents at 25°C and 30°C were significantly higher than those at 20°C. The contents of EGCG and caffeine were the most abundant at 30°C. Theanine content firstly increased and then decreased with the change of temperature, and peaked at 20°C, which was significantly higher than that at 15°C, 25°C and 30°C. Therefore, the temperature of 30°C was optimal for the biosynthesis of EGCG and caffeine, and the temperature of 20°C was optimal for the biosynthesis of theanine.

### 3.2 Tea plants adapt to temperature changes by altering the endogenous hormone levels

The endogenous hormones in tea plants of different groups were discriminately accumulated. (**Figure 1B**; **Supplementary Table 4**). In general, the total content of auxins increased first and then decreased with the temperature went up, and the accumulation of auxins was significantly inhibited at 30°C. Among the components of auxins, the content of IAA-Asp was the highest, and its content was as high as 102.24 ng/g at 25°C, which indicated that the endogenous auxins was mainly accumulated in the form of IAA-Asp in response to temperature changes. As free auxin, IAA content positively responds to temperature, while IBA opposite. The total contents of active gibberellins (GAs) positively respond to temperature changes, but its total amount is lower than other hormones. Interestingly, the contents of GA1, GA4, GAs, tZR and cytokinin (CTK) were the highest at 30°C. The content of tZR was higher than that of other components of CTK at different temperature, which indicated that tZR is the main form of cytokinin in response to temperature, while zeatin content was not significantly changed. The content of ABA negativity responds to temperature changes. The contents of total jasmonic acid (JAs) and total salicylic acid (SAs) increased first and then decreased with the increase of temperature. The contents of free jasmonic acid, free salicylic acid, bound jasmonic acid MeJA, JA-Ile and bound salicylic acid MeSA increased first and then decreased with the increase of temperature, and the contents peaked at 20°C. In terms of the jasmonic acid and salicylic acid, the content of free components was much higher than that of bound components, suggesting that the free JA and SA would be accumulated in the tea plant in response to temperature changes.

### 3.3 Correlation between the content variation of EGCG, theanine, caffeine and endogenous hormones in response to temperature

**Figure 2:**
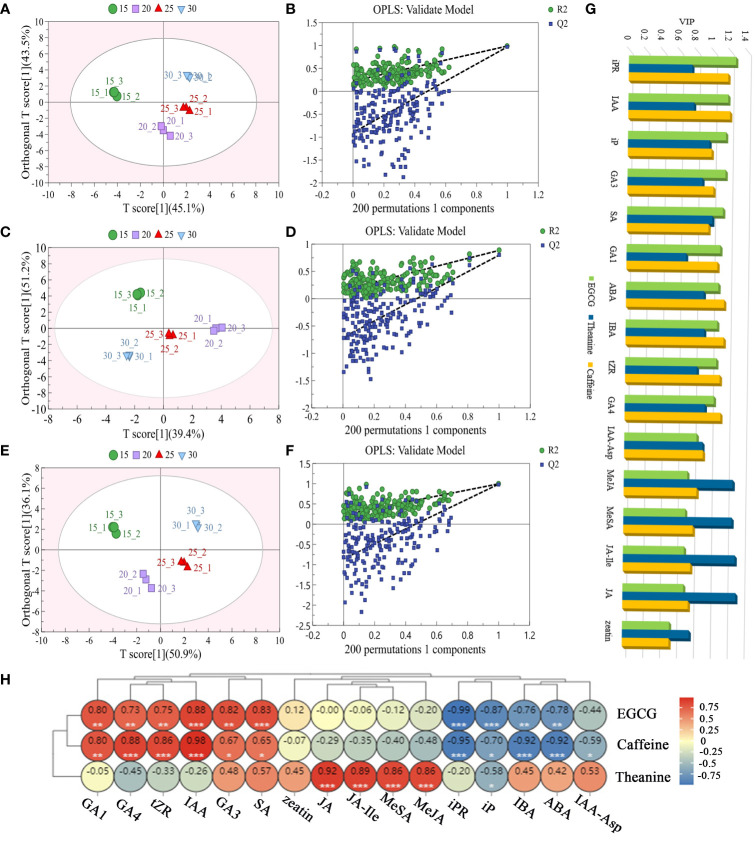
Relationship between the content variations of endogenous hormones and three main taste components in tea plant at different temperature treatments. The score plots **(A, C, E)** and the cross-validation plot **(B, D, F)** of EGCG, theanine, caffeine and 16 endogenous hormones respectively. VIP values **(G)** and Pearson correlation analysis **(H)** of EGCG, theanine, caffeine and 16 hormones contents in tea plant at different temperatures. * Significance level 5% (P < 0.05), ** Significance level 1% (P < 0.01), *** Significance level 0.1% (P < 0.001).

The OPLS analysis was carried out based on the endogenous hormone profiles in the tea samples (**Figure 2**; **Supplementary Table 5**). In the OPLS model of 16 endogenous hormones and EGCG, the model had a 96.3% explanation for the change of endogenous hormone content with temperature, and a 98.9% explanation for the change of EGCG content with temperature, and Q2 = 0.971 (>0.5), indicating that this model had a good predictive ability. As shown in **Figure 2A**, the abundance of EGCG and 16 endogenous hormones in samples treated at different temperatures was significantly different, and different groups could be clearly distinguished. The variable importance of the projection (VIP) values of iPR, IAA, iP, GA3, SA, GA1, ABA, IBA, tZR and GA4 were greater than 1 (VIP>1), indicating that EGCG content was strongly correlated with auxin, gibberellin, cytokinin, abscisic acid and salicylic acid content.

In 16 endogenous hormones and theanine OPLS model, the model had a 90.6% explanation for the change of endogenous hormone content with temperature, and an 88.5% explanation for the change of theanine content with temperature, and Q2 was 0.797 (>0.5), indicating that the model had a good predictive ability. The OPLS analysis showed that 16 endogenous hormones and theanine varied greatly in abundance in samples treated at different temperatures, which could be clearly distinguished at different groups (**Figure 2C**). According to VIP values, the content of JA, JA-Ile, MeJA, SA and MeSA (VIP>1) were highly correlated with theanine content affected by temperature.

In the 16 endogenous hormones and caffeine OPLS model, the model had a 96.4% explanation for the change of endogenous hormone content with temperature, and 99.5% explanation for the change of caffeine content with temperature, and Q2 was 0.987 (>0.5), indicating that the model had a good predictive ability. As shown in **Figure 2E**, the abundance of 16 endogenous hormones and caffeine in samples treated at different temperatures was significantly different, and different groups could be clearly distinguished. According to VIP, the content of IAA, iPR, ABA, IBA, GA4, tZR, GA1, GA3 and caffeine were strongly correlated with temperature (VIP>1).

As showed in Pearson correlation analysis, IAA, SA, GA3, GA1, iPR and iP had strongly related to EGCG with the temperature went up (|r|>0.80, *P*<0.01). However, the relationship between the endogenous hormones of JA, JA-Ile, MeJA, MeSA and theanine content were strong positive correlated with the temperature changed (r>0.85, *P*<0.01). IAA, GA4, tZR, GA1, iPR, IBA, ABA had a powerful correlation with caffeine followed the temperature increasing (|r|>0.80, *P*<0.01), (**Figure 2H**).

In conclusion, under the influence of temperature, iPR, IAA, iP, GA3, SA and GA1 may be the main hormone types affecting EGCG biosynthesis. MeJA, JA-Ile, JA and MeSA may be the chief hormone types that affect theanine synthesis. IAA, iPR, ABA, IBA, GA4, tZR and GA1 may be the principal hormone types that affect caffeine synthesis.

### 3.4 Molecular mechanism of the biosynthesis of main taste components and endogenous hormones in response to temperature

#### 3.4.1 GO and KEGG analysis of DEGs obtained by high-throughout sequencing

**Figure 3:**
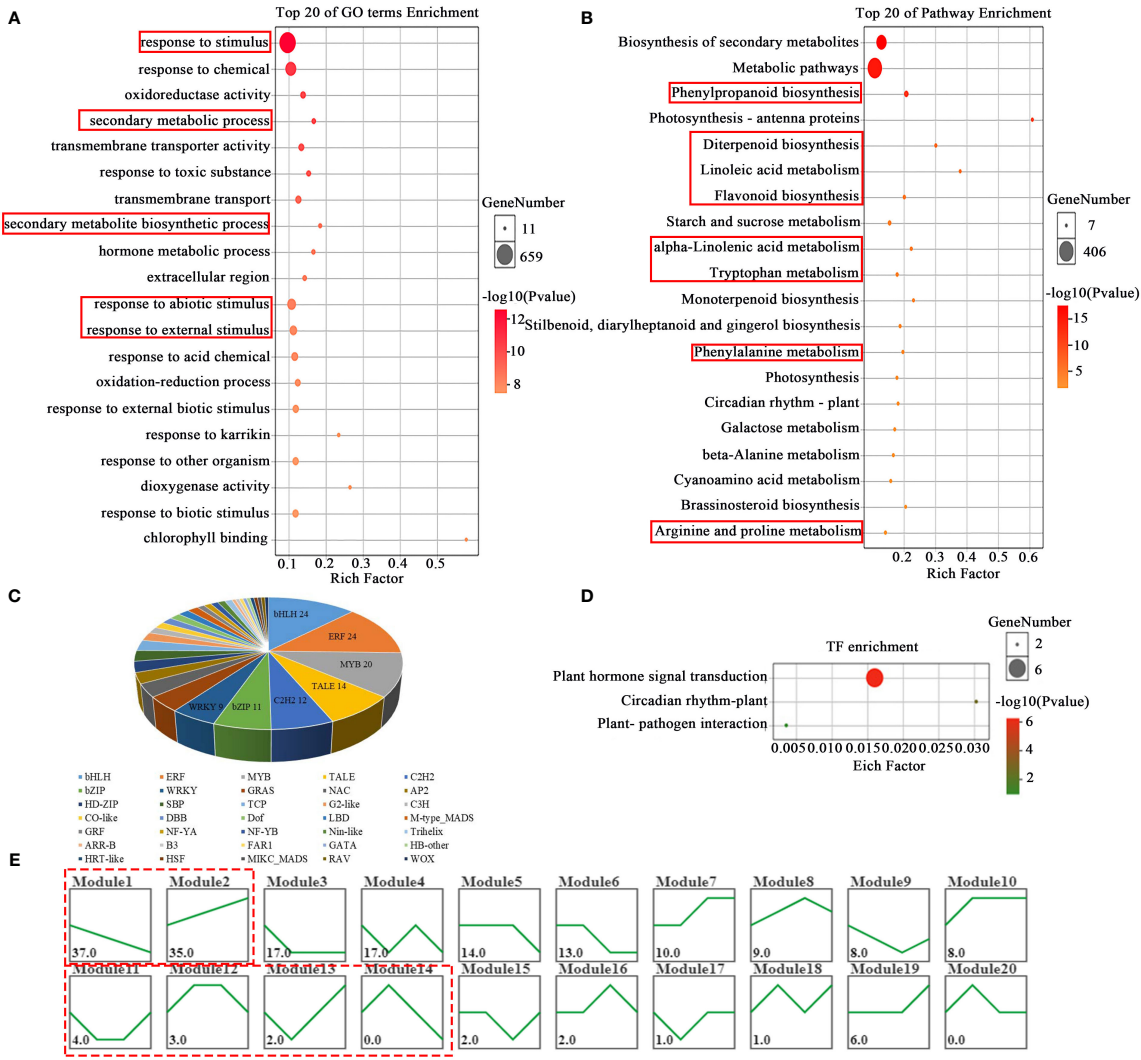
GO and KEGG enrichment analysis and TF prediction of DEGs in bud and one leaf of tea plant at different temperature treatments. The enriched GO **(A)** and KEGG **(B)** pathway of DEGs. Transcription factor prediction **(C)** of DEGs. The enriched KEGG pathway **(D)** of TFs. The expression trend analysis **(E)** of DEGs.

High-throughput RNA-sequencing analysis was performed to detected gene expression profiles of one leaf and a bud samples of tea shoots under different temperature treatments. A total of 43779 genes, and 2590 differentially expressed genes (DEGs, log_2_|FC|>1, *P*<0.05) were obtained. GO enrichment analysis of DEGs (**Figure 3A**) showed that DEGs were mainly enriched in response to stimulus, secondary metabolic process, hormone metabolic process and response to abiotic stimulus (*P ≤* 0.05) at different temperature treatments. KEGG analysis results showed that DEGs mainly enriched in Phenylpropanoid biosynthesis and Flavonoid biosynthesis, beta-Alanine metabolism, Arginine and proline metabolism and some hormone biosynthesis pathways such as Diterpenoid biosynthesis, alpha-Linolenic acid metabolism, Tryptophan metabolism, Phenylalanine metabolism. (*P*<0.05, **Figure 3B**), suggesting that the response to temperature changes in tea plant by initiating the metabolic pathway of a variety of hormone, flavonoid and amino acid.

A total of 189 transcription factors (TF) were predicted and obtained in 2590 DEGs (**Figure 3C**) by using the platform of transcription factor prediction (PlantTFDB http://planttfdb.gao-lab.org/prediction.php). These TFs belong to 36 families, among which the top seven clades were *bHLH* (24), *ERF* (24), *MYB* (16), *TALE* (14), *C2H2* (12), *bZIP* (11) and *WRKY* (9). The KEGG pathway analysis on the 189 TFs was mainly enriched in plant hormone signal transduction (**Figure 3D**).

The expression trends of 189 TFs were analyzed and divided into 20 modules. There were 2 modules including 72 TFs whose expression trends were similarly to EGCG and caffeine content profiles respectively, while 4 modules contained 9 TFs whose expression trends were similar to theanine content profile in the tea samples (**Figure 3E**).

#### 3.4.2 Molecular mechanism of the biosynthesis of EGCG, theanine and caffeine in response to temperature

**Figure 4:**
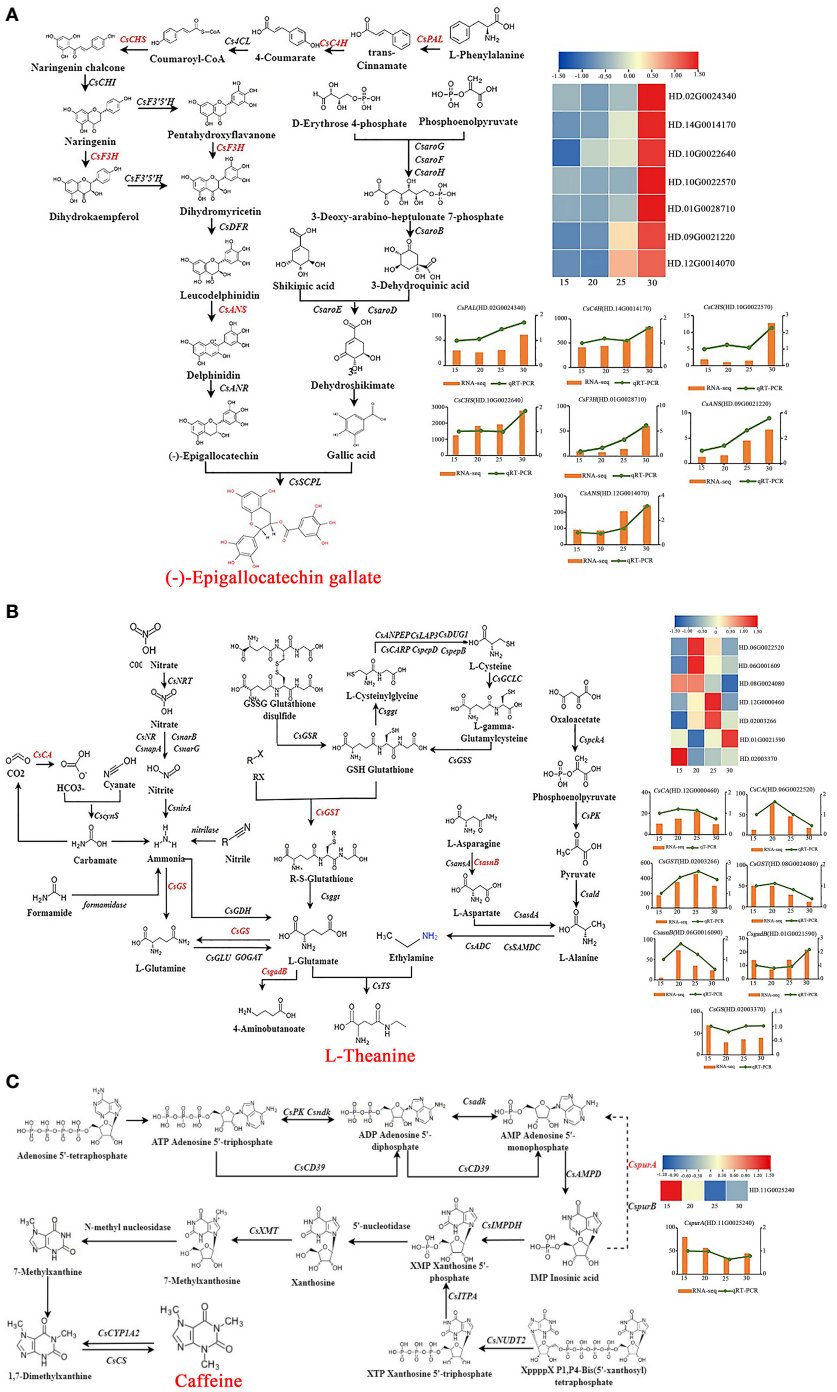
The biosynthesis of EGCG, theanine and caffeine in response to temperature. The biosynthesis of EGCG **(A)**, theanine **(B)** and caffeine **(C)**.

KEGG enrichment analysis showed that 7 genes [*phenylalanine ammonia-lyase* (*CsPAL* (HD.02G0024340)], *CsC4H* (HD.14G0014170), *CsCHS* (HD.10G0022640, HD.10G0022570), *naringenin 3-dioxygenase* [*CsF3H* (HD.01G0028710)] and *CsANS* (HD.12G0014070, HD.09G0021220) were obtained and the expression of these genes were increased with the temperature went up (**Figure 4A**). Pearson analysis showed that the expression of 7 genes were positively correlated with the change of EGCG content. Among them, the correlation between the expression of *CsCHS* (HD.10G0022640), *CsANS* (HD.12G0014070, HD.09G0021220) and *CsC4H* (HD.14G0014170) and the trend of EGCG content was greater than 0.45. It is worth noting that the expression of *CsCHS* (HD.10G0022640) was much higher than that of other genes (FPKM>1000), and there was a significant positive correlation to the trend of EGCG content with temperature increased (r>0.60, *P*<0.05). As a result, 7 genes including *CsPAL* (HD.02G0024340), *CsC4H* (HD.14G0014170), *CsCHS* (HD.10G0022640, HD.10G0022570), *CsF3H* (HD.01G0028710), and *CsANS* (HD.12G0014070, HD.09G0021220) were regulated by temperature to promoted EGCG biosynthesis during tea plant cultivating, and *CsCHS* (HD.10G0022640) was the key gene in regulating the biosynthesis of EGCG at different temperatures.

There were 7 genes enriched in the pathway of theanine biosynthesis. And the biosynthesis of theanine was positively regulated by *CsCA* (HD.06G0022520, HD.12G0000460), *asparagine synthase* [*CsasnB* (HD.06G0016090)], *glutathione S-transferase* [*CsGST* (HD.08G0024080, HD.02003266)], but was negatively regulated by *CsgadB* (HD.01G0021590) and *glutamine synthetase* (*CsGS* (HD.02003370)). There was a significant difference between the expression of *CsCA* (HD.06G0022520) (r>0.60, *P*<0.05), *CsgadB* (HD.01G0021590) (|r|>0.60, *P*<0.05) and theanine content (**Figure 4B**). Therefore, the biosynthesis of theanine was promoted by temperature through reducing the transformation of L-Glutamate to 4-Aminobutanoate *via* increasing the expression of *CsCA* (HD.06G0022520) or down-regulating the expression of *CsgadB* (HD.01G0021590).

It was obviously that the content of caffeine was significantly changed with the increasing of temperature, but there were few genes in response to temperature changes at transcriptional level. As a decomposition gene of inosinic acid (IMP), the expression of *CspurA* (HD.11G0025240) was negatively respond to temperature changes, which was opposite to the trend of caffeine content (**Figure 4C**). The accumulation of caffeine at high temperature is probably ascribed to the downregulating expression of *CspurA* (HD.11G0025240), which inhibited the decomposition of IMP, and increased the content of IMP, thus promoting the biosynthesis of caffeine.

#### 3.4.3 Molecular mechanism of the biosynthesis of endogenous hormones in response to temperature

**Figure 5:**
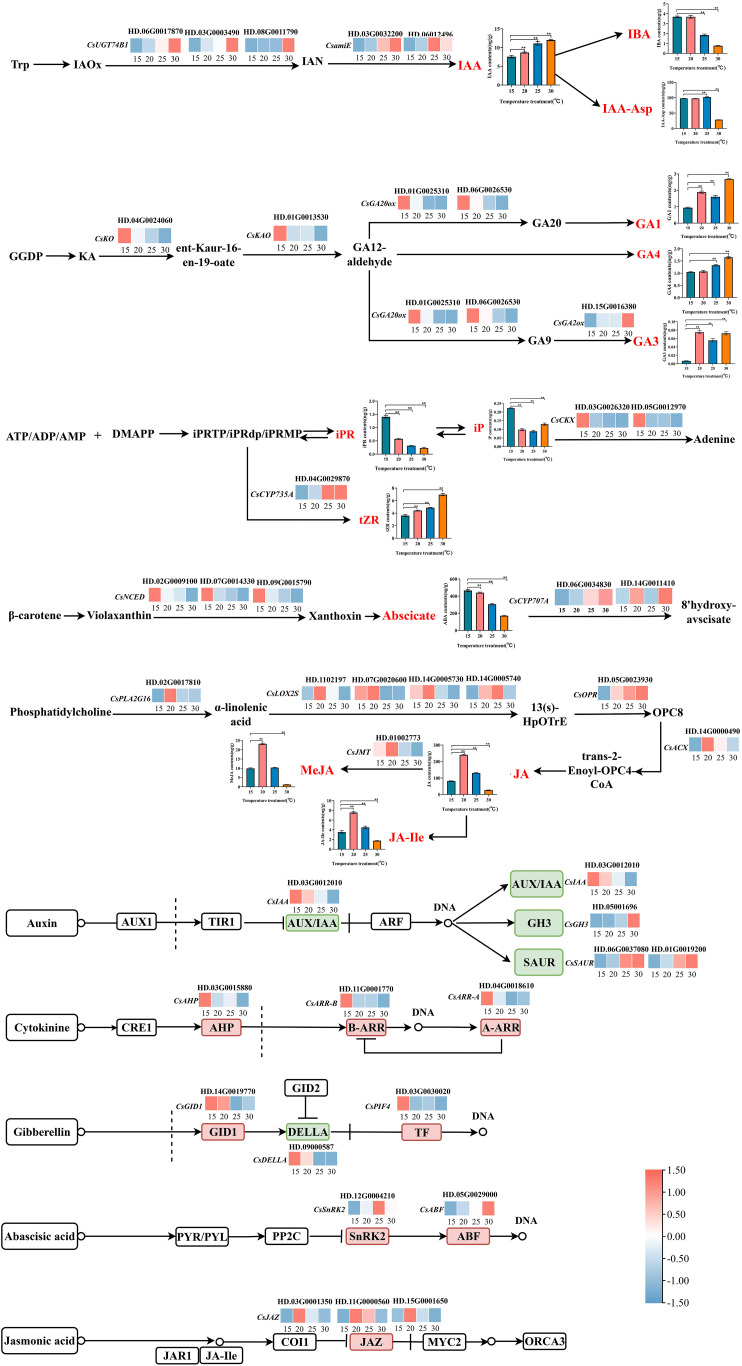
The biosynthesis of endogenous hormones in response to temperature in tea plant. * Significance level 5% (P < 0.05), ** Significance level 1% (P < 0.01).

We performed KEGG functional enrichment analysis on 2590 DEGs. There were 5, 5, 3, 5 and 8 genes screened out as the candidates impacting the biosynthesis of auxin, gibberellin, cytokinin, abscisic acid and jasmonic acid respectively, and 15 genes were involved in the plant hormone signal transduction pathways of these five hormones (**Figure 5**).

The synthesis and accumulation of IAA was promoted by temperature *via* up-regulating the expression of 3 *N-hydroxythioamide S-beta-glucosyltransferase* [*CsUGT74B1* (HD.06G0017870, HD.03G0003490, HD.08G0011790)] and 2 *amidase* [*CsamiE* (HD.03G0032200, HD.06012496)]. However, the accumulations of IBA and IAA-Asp in tea plants were different from that of IAA, indicating that tea plants may respond to temperature by changing the existence form of auxin. Auxin signals were transmitted to downstream target genes through the expression of *CsIAA* (HD.03G0012010), *SAUR family protein* [*CsSAUR* (HD.06G0037080, HD.01G0019200)] and *auxin responsive GH3 gene family* [*CsGH3* (HD.05001696)].

The biosynthesis of GA3 was positively regulated by temperature through upregulating the expression of *gibberellin 2beta-dioxygenase* [*CsGA2ox* (HD.15G0016380)]. On the contrary, *ent-kaurenoic acid monooxygenase* [*CsKAO* (HD.01G0013530)], *ent-kaurene oxidase* [*CsKO* (HD.04G0024060)], *gibberellin-44 dioxygenase* [*CsGA20ox* (HD.01G0025310, HD.06G0026530)] were negatively responsive to temperature changes and opposite to the accumulation trend of GA1, GA3 and GA4. We speculated that this result might be caused by the differential expression of these genes at the protein level. Gibberellin also transmitted its signal to downstream genes by inhibiting *gibberellin receptor GID1* [*CsGID1* (HD.14G0019770)], *CsDELLA* (HD.09000587) and *phytochrome-interacting factor 4* [*CsPIF4* (HD.03G0030020)], and regulated the biosynthesis of downstream metabolites.

For cytokinin, the contents of tZR, iPR and iP responded to temperature changes, but the response patterns were different. The biosynthesis of tZR was affected by temperature through upregulating the expression of *cytokinin trans-hydroxylase* [*CsCYP735A* (HD.04G0029870)]. While iPR and iP negatively respond to temperature changes by regulating the expression of *cytokinin dehydrogenase* [*CsCKX* (HD.03G0026320, HD.05G0012970)], and making cytokinin content to achieve a relatively stable level in tea plant. At different temperature, cytokinin transmitted signals to downstream genes *via histidine-containing phosphotransfer peotein* [*CsAHP* (HD.03G0015880)], *two-component response regulator ARR-A family* [*CsARR-A* (HD.04G0018610)], and *two-component response regulator ARR-B family* [*CsARR-B* (HD.11G0001770)].

The accumulation of ABA was inhibited by temperature through negatively regulating the expression of *9-cis-epoxycarotenoid dioxygenase* [*CsNCED* (HD.07G0014330, HD.02G0009100, HD.09G0015790)] with the temperature increased. Additionally, as a downstream degradation gene of ABA, *(+)-abscisic acid 8’-hydroxylase* [*CsCYP707A* (HD.14G0011410, HD.06G0034830)] is highly expressed at high temperature, thus accelerating the decomposition of ABA at high temperature. At different temperature, ABA mediated the synthesis of downstream metabolites by inhibiting *serine/threonine-protein kinase SRK2* [*CsSnRK2* (HD.12G0004210)] and *ABA responsive element binding factor* (*CsABF* (HD.05G0029000)).

In this study, the accumulations of JA, JA-Ile and MeJA were consistent along with the variation of temperature, and which peaked at 20 °C. These observations might be associated with the expressions of *HRAS-like suppressor 3* [*CsPLA2G16* (HD.02G0017810)], *lipoxygenase* [*CsLOX2S* (HD.1102197, HD.07G0020600, HD.14G0005730, HD.14G0005740)], *12-oxophytodienoic acid reductase* [*CsOPR* (HD.05G0023930)] and *acyl-CoA oxidase* [*CsACX* (HD.14G0000490)], which were initiated to regulate the biosynthesis of JA. Moreover, the high expression of *jasmonate O-methyltransferase* [*CsJMT* (HD.01002773)] accelerated the degradation of JA and promoted the biosynthesis of MeJA. At different temperatures, the JA signal regulated the downstream genes through *CsJAZ* (HD.03G0001350, HD.11G0000560, HD.15G0001650).

### 3.5 Mechanism of the regulation on biosynthesis of EGCG, theanine and caffeine by hormone at different temperatures

#### 3.5.1 The networks of correlation between hormone signal transduction factors, transcription factors and structural genes related to the biosynthesis of main taste components at different temperatures

**Figure 6:**
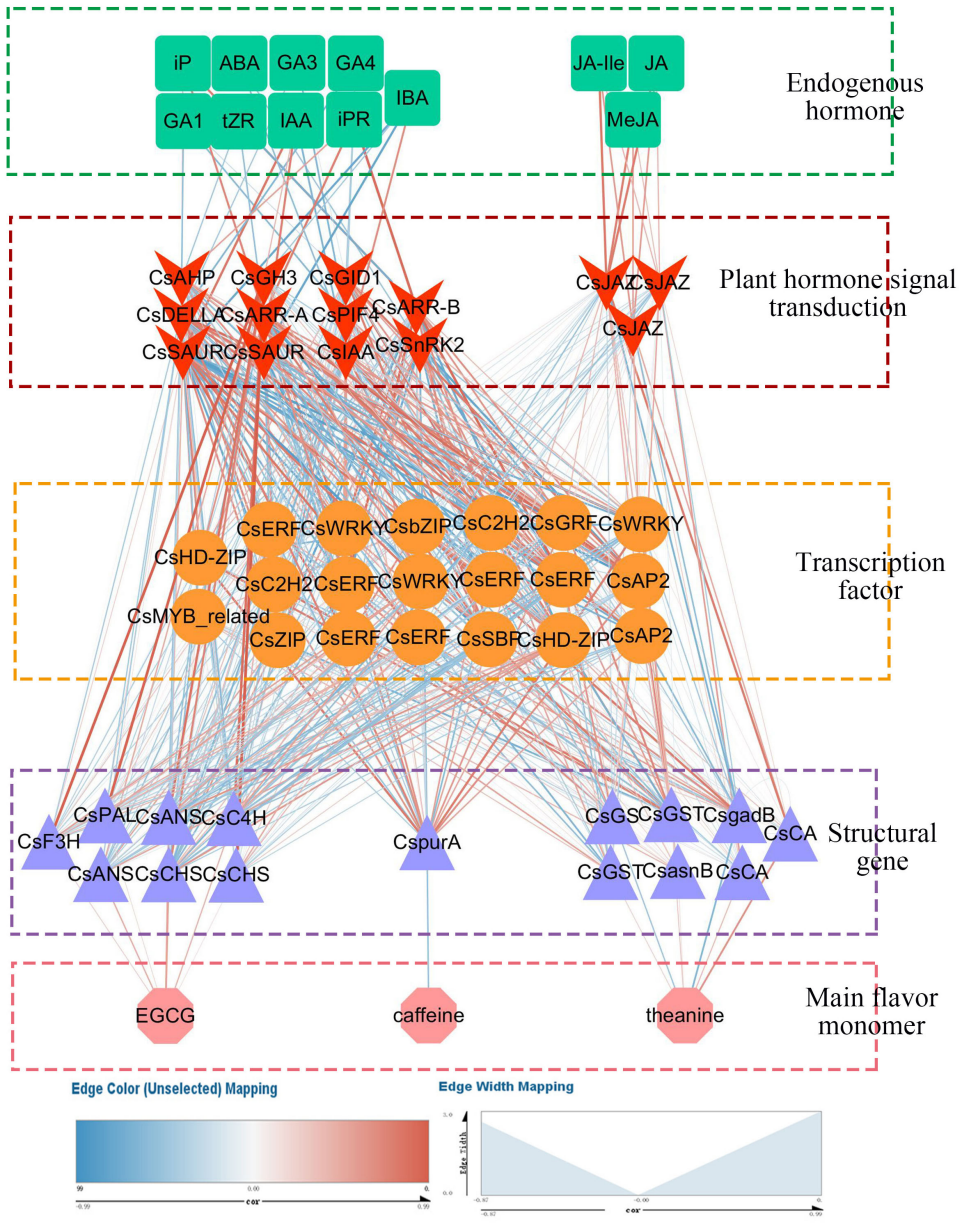
The networks of endogenous hormone, EGCG, theanine and caffeine contents and their associated gene in tea plants at different temperatures.

The online software JASPAR (https://jaspar.genereg.net/) was used to predict 81 TF binding sites. The results showed that a total of 20 transcription factors had binding sites with 15 structural genes in the biosynthesis pathways of EGCG, theanine and caffeine (Score threshold>0.80). The correlation analysis was conducted on the selected hormones, their signal transduction factors, transcription factors and structural genes, and the results were drawn into a network diagram (**Figure 6**).

For EGCG, 4 structural genes namely *CsC4H* (HD.14G0014170), *CsCHS* (HD.10G0022640), *CsANS* (HD.09G0021220, HD.12G0014070) were positively correlated with the content of EGCG. Auxin signal transduction factor *CsIAA* (HD.03G0012010) was negatively correlated with *CsCHS* (HD.10G0022640) (r>0.60, *P*<0.05). The gibberellin signal transduction factor *CsDELLA* (HD.09000587) was negatively correlated with *CsC4H* (HD.14G0014170) and *CsANS* (HD.09G0021220) (|r|>0.60, *P*<0.05). It is worth noting that auxin signal transduction factor *CsSAUR* (HD.01G0019200, HD.06G0037080) and cytokinin signal transduction factor *CsARR-A* (HD.04G0018610), *CsARR-B* (HD.11G0001770) had strong correlation with *C2H2 family protein* [*CsC2H2* (HD.04G0027010)], *Squamosa promoter binding protein* [*CsSBP* (HD.04G0020310)), *basic (region-leucine)zipper family protein* [*CsbZIP* (HD.05G0001910)], *ethylene responsive element binding factor* [*CsERF* (HD.06G0016330, HD.06G0016380)], *Homeodomain-leucinezipper* [*CsHD-ZIP* (HD.05G0006810)], *WRKY DNA-binding protein* [*CsWRKY* (HD.09G0023240)], *growth-regulating factor* [*CsGRF* (HD.11G0012770)] (0.60<|r|<0.91, *P*<0.05). All of *CsC2H2* (HD.04G0027010), *CsSBP* (HD.04G0020310), *CsbZIP* (HD.05G0001910), *CsERF* (HD.06G0016330, HD.06G0016380) and *CsWRKY* (HD.09G0023240) had strong correlation with *CsIAA* (HD.03G0012010) (|r|>0.60, *P*<0.05). Gibberellin signal transduction factor *CsPIF4* (HD.03G0030020) was positively correlated with *CsERF* (HD.06G0016330, HD.06G0016380) and *CsWRKY* (HD.09G0023240) (r>0.64, *P*<0.05), and *CsDELLA* (HD.09000587) was positively correlated with *CsSBP* (HD.04G0020310), *CsERF* (HD.06G0016330), *CsWRKY* (HD.09G0023240), *CsGRF* (HD.11G0012770) (r>0.57, *P*<0.05). Structural genes of *CsC4H* (HD.14G0014170), *CsCHS* (HD.10G0022640), *CsANS* (HD.09G0021220, HD.12G0014070) on EGCG biosynthesis positively correlated with *CsbZIP* (HD.05G0001910) and *CsHD-ZIP* (HD.05G0006810), and negatively correlated with *CsC2H2* (HD.04G0027010), *CsSBP* (HD.04G0020310), *CsERF* (HD.06G0016330, HD.06G0016380), *CsWRKY* (HD.09G0023240) and *CsGRF* (HD.11G0012770).

As for theanine, the expression of *CsCA* (HD.06G0022520) and *CsgadB* (HD.01G0021590) were significantly correlated with theanine content (|r|>0.60, *P*<0.05). The correlation of *CsJAZ* (HD.03G0001350, HD.15G0001650) with *CsCA* (HD.06G0022520), *CsgadB* (HD.01G0021590) were more than 0.50. And the pairwise correlations between *CsJAZ* (HD.03G0001350, HD.15G0001650), *CsWRKY* (HD.07G0017570), *CsAP2* (HD.10G0007680) and *CsgadB* (HD.01G0021590) were greater than 0.40.

At different temperature, the expression of *CspurA* (HD.11G0025240) was negatively correlated with caffeine content (|r|<0.60, *P*>0.05). There was a strongly negative correlation between *CsSnRK2* (HD.12G0004210) and *CspurA* (HD.11G0025240) (|r|>0.60, *P*<0.03). The hormone signal factors namely *CsIAA* (HD.03G0012010), *CsSAUR* (HD.01G0019200), *CsDELLA* (HD.09000587), *CsARR-A* (HD.04G0018610), *CsARR-B* (HD.11G0001770), *CsABF* (HD.05G0029000), *CsSnRK2* (HD.12G0004210) were correlated with *CsC2H2* (HD.02G0015370), *CsHD-ZIP* (HD.05G0010130), *CsWRKY* (HD.08002776), *CsWRKY* (HD.09G0023240) and *CsGRF* (HD.11G0012770) (0.30<|r|<0.90), *CsPIF4* (HD.03G0030020) was significantly related to *CsWRKY* (HD.09G0023240) (|r|>0.50, *P*<0.05). The relationship between this 5 TFs and *CspurA* (HD.11G0025240) had strong correlation (r>0.60, *P*<0.05).

Overall, hormones and their signal transduction factors were correlated with transcription factors and structural genes related to the biosynthesis of main taste components in tea plant at different temperatures.

#### 3.5.2 Hormones affect the biosynthesis of main taste components mediated by signal transduction factors at different temperatures

**Figure 7:**
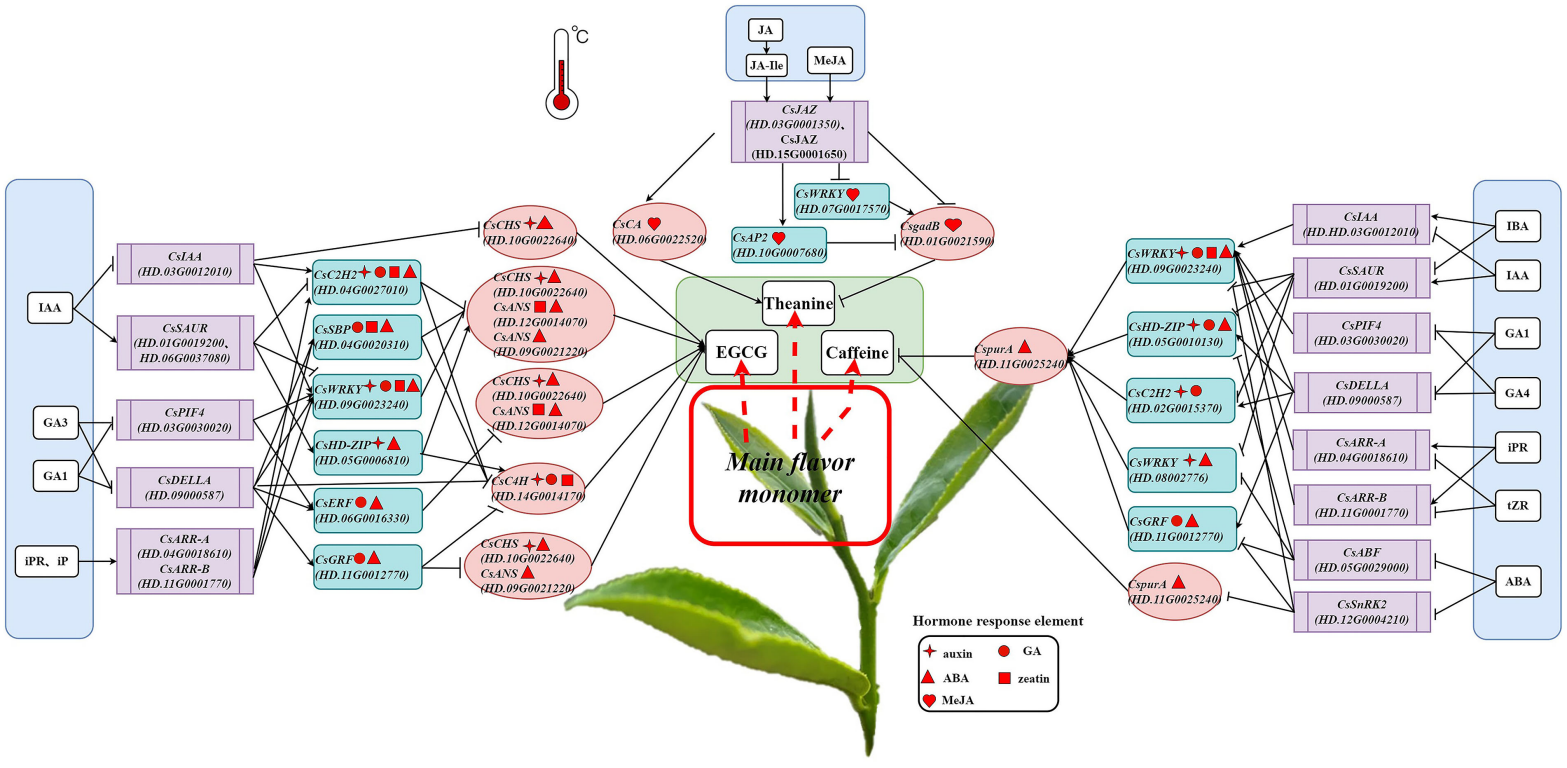
Mechanisms of the biosynthesis of EGCG, theanine and caffeine in response to temperature mediated by hormone signal transduction factors.

PlantCARE (http://bioinformatics.psb.ugent.be/webtools/plantcare/html/) was used to predict the promoters of 20 transcription factors and 7 structural genes of main taste components synthesis related genes, and various hormone response elements on the promoters were screened (**Supplementary Table 6**). Based on the results of correlation analysis, transcription factor binding site and promoter prediction analysis, we speculated that under different temperature cultivation conditions, endogenous hormones can mediate signal transduction factors to directly regulate the expression of structural genes related to EGCG, theanine and caffeine biosynthesis in tea plants. It may also mediate transcription factors to initiate the expression of downstream EGCG, theanine and caffeine biosynthesis-related target genes, thus affecting the synthesis and accumulation of these three main taste components in tea plants (**Figure 7**).

Possible pathways of temperature influences on EGCG biosynthesis through hormones: IAA regulated EGCG biosynthesis by directly mediating the expression of *CsCHS* through its signal transduction factor *CsIAA*; GA1 and GA3 promoted the expression of *CsC4H* and regulated EGCG synthesis by inhibiting *CsDELLA*; IAA, iPR and iP could also mediate *CsC2H2* to regulate the expression of *CsC4H*, *CsCHS* and *CsANS* and control the biosynthesis of EGCG through their signal transduction factors *CsIAA*, *CsSAUR*, *CSARR-A*, and *CSARR-B*, respectively; GA1 and GA3 inhibited the expression of *CsERF* and *CsGRF* through the signal transduction factor *CsDELLA*, and initiated the expression of downstream genes *CsCHS* and *CsANS* to promote EGCG biosynthesis; It is noteworthy that all of IAA, GA1, GA3, iPR and iP acted on *CsWRKY* through signal transduction factors *CsIAA*, *CsSAUR*, *CsPIF4*, *CsDELLA*, *CsARR-A* and *CsARR-B* respectively, and regulated the expression of structural genes *CsCHS* and *CsANS* to affect the EGCG biosynthesis.

The biosynthesis of theanine might be promoted by temperature changes in two ways. First, JA, JA-Ile and MeJA promoted the synthesis of theanine by promoting *CsCA* and inhibiting the expression of *CsgadB* through the signal transduction factor *CsJAZ*; another way, JA, JA-Ile and MeJA could also inhibit *CsWRKY* and promote *CsAP2* through *CsJAZ* to down-regulate the expression of *CsgadB* and promoted the synthesis of theanine.

Possible mechanisms of temperature affects caffeine biosynthesis through hormones: IAA, IBA, GA1, GA4, tZR, iPR and ABA mediated *CsWRKY* to regulate the expression of *CspurA* through their signal transduction factors *CsIAA*, *CsSAUR*, *CsPIF4*, *CsDELLA*, *CsARR-A*, *CsARR-B* and *CsSnRK2*, respectively, thus affecting the synthesis of caffeine; IAA, IBA, GA1, GA4 and ABA respectively mediated *CsHD-ZIP* to regulate the expression of downstream gene *CspurA* through their signal transduction factors *CsSAUR*, *CsDELLA* and *CsABF*, which affected the synthesis of caffeine; IAA, GA1 and GA4 mediated *CsC2H2* to down-regulated the expression of *CspurA* and promoted the synthesis of caffeine through *CsSAUR* and *CsDELLA*, respectively; GA1 and GA4 mediated *CsGRF* to down-regulate the expression of *CspurA via* the signal transduction factor *CsDELLA* which promoted the synthesis of caffeine; ABA promoted the expression of *CsGRF* to up-regulate the expression of *CspurA* and inhibited the biosynthesis of caffeine through *CsABF* and *CsSnRK2*, respectively.

### 3.6 Verification of related genes by qRT-PCR

To verify the reliability of the HTS RNA-Seq data, we performed qRT-PCR to validate the expression profiles of the 36 core genes obtained by screening. Expression profiles under different temperature were compared by qRT-PCR. For all these selected genes, the expression trends by qRT-PCR were basically consistent with those of the HTS RNA-Seq data, which proved that the HTS RNA-Seq data were reliable (**Figure 4**; **Supplementary Figure 1**).

## 4 Discussion

In our study, we found that IAA, GA1 and GA3 were significantly correlated with EGCG content, JA, JA-Ile and MeJA were strongly correlated with theanine content, and IAA, GA1 and GA4 were significantly correlated with caffeine content at different temperatures. We speculate the regulatory mechanisms of the biosynthesis of EGCG, theanine and caffeine in response to temperature mediated by hormone signal transduction factors.

Exogenous spraying of JA, SA, MeSA, BR melatonin and ABA showed that different hormones had effects on the contents of flavonoids (Li et al., 2019b), polyphenols (Li et al., 2016), anthocyanin (Gai et al., 2020), theaflavin (Liao et al., 2019), lignin (Liu et al., 2023), catechin (Liao et al., 2019), amino acid (Li et al., 2016), theanine (Li et al., 2020) and caffeine (Liao et al., 2019) of tea plant. Under biotic and abiotic stress, JA, BR and ABA were related to resistance to drought (Zhou et al., 2014), against *Colletotrichum gloeosporioides* (Zhang et al., 2018), invasion of tea geometrids (Zhang et al., 2020b). Based on the results of a variety of bioinformatics analysis and multidimensional correlation analysis, we elucidated the molecular mechanism of temperature regulation on the main taste components and on endogenous hormones of tea plant (**Figure 4**, **5**), and also conjecture the regulatory mechanisms of the biosynthesis of EGCG, theanine and caffeine in response to temperature mediated by hormone (**Figure 7**).

### 4.1 The biosynthesis of EGCG was affected by temperature *via* IAA, GA1, GA3, iPR and iP

Our results showed that the contents of IAA, GA1, GA3, iPR and iP were significantly correlated with the content of EGCG. The synthesis of EGCG was promoted at 30 °C, which was consistent with our previous study (Xiang et al., 2021a). From the perspective of temperature, it is confirmed that the EGCG content of tea planted in greenhouse is higher than that of field planting, and the EGCG content of tea in autumn is higher than that in spring (Liu et al., 2015a; Liu et al., 2015b). *CHS* and *ANS*, as key genes in EGCG synthesis pathway, directly affect the biosynthesis of EGCG (Yin et al., 2020). Previous studies have found that exogenous application of IAA can increase the expression of CHS protein (Luo et al., 2016). Our results showed that higher temperature promoted the biosynthesis of IAA, and conjecture that IAA upregulated the expression of *CsCHS* at the transcriptional level through its signal transduction factor *CsIAA* promoting the synthesis of EGCG. Zhang and Li also found that two *C2H2-type zinc finger proteins* (*CsC2H2-ZFPs*) were significantly correlated with the content of EGCG, and *C2H2* and *SBP* were significantly correlated with the content of total flavonoids (Li et al., 2021; Zhang et al., 2021), which was consistent with our results. It has been confirmed that ERF11 interacts with RGA protein to enhance GA signal in *Arabidopsis thaliana* (Zhou et al., 2016). The interaction between WRKY45 and DELLA protein RGL1 inhibited the transcriptional activation activity of WRKY45 and weakened the expression of downstream target genes (Chen et al., 2017). Overexpression of *MdWRKY11* in apple callus promoted the expressions of *F3H*, *FLS*, *DFR*, *ANS* and *UFGT*, and increased the accumulation of flavonoids and anthocyanins (Wang et al., 2018). These results are consistent with the inference of this study that *CsDELLA* interacts with *CsERF* and *CsWRKY*, and *CsANS* is a target gene of *CsWRKY*. However, there are differences in the direction of regulation, which may be caused by differences in different species or different gene members in the same family. The mechanism needs further study. Based on the results of our study, we also obtain the speculations: GA1 and GA3 directly mediated the expression of *CsC4H* through *CsDELLA* and promoted the synthesis of EGCG; IAA promoted the expression of *CsCHS* and *CsANS* through *CsIAA* and *CsSAUR* mediated *CsWRKY*, and increased the content of EGCG; iPR and iP inhibited EGCG biosynthesis through *CsARR-A* and *CsARR-B* mediated *CsC2H2*, *CsSBP* and *CsWRKY*, and the protein interaction needs to be further verified.

### 4.2 The biosynthesis of theanine was affected by temperature *via* JA, JA-Ile and MeJA

The results showed that the optimum temperature for theanine biosynthesis was 20°C, and it was mainly regulated by jasmonic acid signaling pathway. Previous studies have found that the synthesis and accumulation of theanine in tea plants increased in spring, while the content of theanine decrease in winter (Li et al., 2019a) which was caused by temperature changes in different season. High or low temperatures could reduce theanine content levels (Li et al., 2018; Li et al., 2020; Huang et al., 2021). These results further confirmed the conclusion of our study that higher or lower temperature adverse to increase the synthesis and accumulation of theanine. Zhao also found that there is a positive correlation between JA and theanine content (Zhao et al., 2020), and JAZ protein could interact with GhWRKY22 protein (Wang et al., 2019). These conclusions are consistent with our findings that JA, JA-Ile and MeJA may directly promote *CsCA* or inhibit the expression of *CsgadB* through *CsJAZ*, or reduce the expression of *CsgadB* by inhibiting *CsWRKY* to promote the synthesis of theanine.

### 4.3 The biosynthesis of caffeine was affected by temperature *via* IAA, IBA, GA1, GA4 and iPR

Caffeine content positively responded to the change of temperature. According to the results of our study, it is speculated that auxin, gibberellin, cytokinin and abscisic acid affected the caffeine biosynthesis by mediating *CsWRKY* to regulate the expression of *CspurA* through their signal transduction factors, respectively. Previous studies found that IAA and ZA were positively correlated with caffeine content (Zhao et al., 2020), and it is consistent with the results of our study. An interaction relationship between *DELLA* and *WRKY* has been demonstrated in *Arabidopsis thaliana* (Chen et al., 2017), which provides evidence for our inferences that GA1 and GA4 mediated *CsWRKY* down-regulated the expression of downstream gene *CspurA* through signal transduction factor *CsDELLA*, and promoted the synthesis of caffeine. We also presume that iPR inhibited caffeine biosynthesis by promoting *CspurA* expression *via* its signal transduction factors *CsARR-A* and *CsARR-B* mediated the expression of *CsWRKY*, and the interaction mechanism needs to be further verified.

## 5 Conclusion

In conclusion, the biosynthesis of EGCG, theanine and caffeine in tea plant in response to temperature is mediated by endogenous hormone signal transduction factors. *CsCHS* and *CsANS* expression of EGCG biosynthetic genes in response to temperature mediated by hormone signal transduction factors *CsIAA* and *CsDELLA*. *CsCA* and *CsgadB* expression of theanine biosynthetic genes in response to temperature mediated by jasmonic acid signal transduction factor *CsJAZ*. *CspurA* expression of caffeine biosynthetic gene in response to temperature mediated by hormone signal transduction factor *CsIAA* and *CsDELLA via* transcription factor *CsWRKY*. These results provide a new perspective for the biosynthesis of main flavor components regulated by temperature, and provide a basis for improving the quality of fresh tea leaves.

## Data availability statement

The datasets presented in this study can be found in online repositories. The names of the repository/repositories and accession number(s) can be found below: https://www.ncbi.nlm.nih.gov/, PRJNA925799.

## Author contributions

QZ conducted an experiment, and analyzed the data, and wrote a manuscript. LL, XL, XD, BC, MT, JH, LJW conducted an experiment. WK analyzed the data. QZ, PX, LYW and JL participated in the experimental design and guided the research. LYW, YS and JL reviewed the manuscript. All authors contributed to the article and approved the submitted version.
