# Supplementary material for: The biosynthesis of EGCG, theanine and caffeine in response to temperature is mediated by hormone signal transduction factors in tea plant (Camellia sinensis L.)
Source: Front Plant Sci. 2023 Mar 23;14:1149182. doi: 10.3389/fpls.2023.1149182 (PMC10076774; doi:10.3389/fpls.2023.1149182)
Supplement: Supplementary file 1 [file DataSheet_1.docx]

Supplementary Material

# Supplementary Table

**Supplementary Table 1** RNA-Seq quality statistics

| **Sample** | **Total Raw Reads (M)** | **Total Clean Reads (M)** | **Total Clean Bases (Gb)** | **Clean Reads Q20 (%)** | **Clean Reads Q30 (%)** | **Clean Reads Ratio (%)** |
| --- | --- | --- | --- | --- | --- | --- |
| 15_1 | 47.33 | 42.51 | 6.38 | 95.69 | 89.98 | 89.82 |
| 15_2 | 47.33 | 41.97 | 6.3 | 95.98 | 90.65 | 88.68 |
| 15_3 | 47.33 | 42.51 | 6.38 | 95.95 | 90.55 | 89.83 |
| 20_1 | 47.33 | 42.05 | 6.31 | 95.81 | 90.28 | 88.86 |
| 20_2 | 47.33 | 41.99 | 6.3 | 95.81 | 90.25 | 88.73 |
| 20_3 | 47.33 | 42.06 | 6.31 | 95.67 | 89.94 | 88.87 |
| 25_1 | 49.08 | 43.43 | 6.52 | 96.52 | 91.73 | 88.5 |
| 25_2 | 47.33 | 42.74 | 6.41 | 96.35 | 91.33 | 90.32 |
| 25_3 | 47.33 | 42.76 | 6.41 | 96.35 | 91.31 | 90.35 |
| 30_1 | 47.33 | 43.26 | 6.49 | 96.38 | 91.31 | 91.41 |
| 30_2 | 47.33 | 42.89 | 6.43 | 96.35 | 91.3 | 90.63 |
| 30_3 | 47.33 | 43.15 | 6.47 | 96.38 | 91.37 | 91.18 |

**Supplementary Table 2** Primer sequence for qRT-PCR

| **Gene Family** | **Gene ID** | **primer-F** | **primer-R** |
| --- | --- | --- | --- |
| *Csβ-Actin* | HD.01G0021100 | TGAGGATATTCAGCCCCTTG | GCCTGGGTCGACCTACAATA |
| *CsPAL* | HD.02G0024340 | GAGGCGAGACCTTGACCATA | CCCCTTATTCATGCTCTCCA |
| *CsC4H* | HD.14G0014170 | AGGCTAAGGTTGAGGCCAAT | GGCAAGAGCTCGAAATTCTG |
| *CsCHS* | HD.10G0022640 | GGCACAGTACTTCGCCTAGC | TCACCAAATAGGGCTTGACC |
| *CsCHS* | HD.10G0022570 | CATGGTTGTGGTTGAAGTGC | GCAAAAGACCAGGTGGGTAA |
| *CsF3H* | HD.01G0028710 | CAAGGTCGCCTACAACCAAT | TCACACGCTTCGACAATCTC |
| *CsANS* | HD.09G0021220 | GGGAGCAAATTGGCTAACAA | TCAGCTGTTGCGGAATGTAG |
| *CsANS* | HD.12G0014070 | CAGGTTCCCACAGTGGACTT | TATCCCGTGGTTCACCAAAT |
| *CsCA* | HD.06G0022520 | AACTTGCTGAAGGCCAGAAA | GTTAAATCCCGGGACCAAGT |
| *CsCA* | HD.12G0000460 | TCGTTGTAGCCTCTGCATTG | TGCCGGAATAACTGAACACA |
| *CsGS* | HD.02003370 | TCAAGTTGGACCTGCTGTTG | CTCGATAGGTTTGGGGTCAA |
| *CsGST* | HD.08G0024080 | TCTTGAGATGAACCCGATCC | GGCAACAACGAAGCTTTCTC |
| *CsGST* | HD.02003266 | ATGCAGACTACGGTCCCAAC | ACCACCTGCTGAAGAACCAG |
| *CsasnB* | HD.06G0016090 | GGGCCTTTGATGATGAAGAA | TTTAAGCCCGTCAATCCAAC |
| *CsgadB* | HD.01G0021590 | CTAAATTTGGCGTCGTTCGT | CTCGGTGGTGACAGGGTACT |
| *CspurA* | HD.11G0025240 | TTTGCCCTTCATCTTGTTCC | TTTGCAGGAGACCCCATTAG |
| *CsIAA* | HD.03G0012010 | GCTTTACAATCGGGCAATGT | ACATCACCAACAAGCATCCA |
| *CsSAUR* | HD.01G0019200 | AAAGGCCCCATCAATATTCC | GCAAAGAAGATGGTGGTGGT |
| *CsSAUR* | HD.06G0037080 | CCAATCTCGAGGATTTCCAA | ACTTGTCGGTAAGCCCATGA |
| *CsPIF4* | HD.03G0030020 | ATGGCTAAGCGACTTGAGGA | CCAGAACACGCAGATGAAGA |
| *CsDELLA* | HD.09000587 | ATCACGTCAAAGGGTTCAGG | TTCCAGAGAATTGGCAAACC |
| *CsARR-B* | HD.11G0001770 | TTAGGGAATGGCTTCAATCG | TCATTTCAATGCATGCTGGT |
| *CsARR-A* | HD.04G0018610 | AGGGTTCGGATCATCTGTTG | GGCCGTAACTCTGCAAGAAG |
| *CsABF* | HD.05G0029000 | CGATCACGAGGCCAAATAAT | TGCTGAAACCCAAATGTCAA |
| *CsJAZ* | HD.03G0001350 | AAGTTCATTGATCGCCGAAG | GTGTGATCGACAGAGCCAGA |
| *CsJAZ* | HD.15G0001650 | TCAAATTTTGCGCTGACTTG | TTCTTGTGGTTTCGGGTCTC |
| *CsGRF* | HD.11G0012770 | CCTCTCATTGATCCCTTCCA | ATCGAAGAAATGGCGAAGAA |
| *CsC2H2* | HD.04G0027010 | TTGCTTCAGAAAGCTGCTCA | CCTTCCAATCAGAGCCACTC |
| *CsSBP* | HD.04G0020310 | TCTCGGGTTCTTTGATCCAC | CCACAGCCTGACATCCCTAT |
| *CsHD-ZIP* | HD.05G0006810 | GGCAAGGACAAAGTTGAAGC | GCTCATTCACCTCCTTCTGC |
| *CsERF* | HD.06G0016330 | GACCCATCACCAAAATCACC | CTTTCGTGGTTCGCGTATTT |
| *CsWRKY* | HD.09G0023240 | GCTGGGCTACAAAGCAAGTC | GGTGGTGGTAGTGGAGCTGT |
| *CsC2H2* | HD.02G0015370 | ACCTCCCAGGAACTCCAGAT | CTCTGAAACCCTTTCCCACA |
| *CsWRKY* | HD.08002776 | CAAGAGCATTTGGCTTGTCA | GTTTGGGGTAACCGGAGTTT |
| *CsHD-ZIP* | HD.05G0010130 | ACACCCTCAATCCCAAACAA | CTCACAATCCACCTCCGTTT |
| *CsWRKY* | HD.07G0017570 | ATGTTGATGGGACGGTGATT | TCCCACACCATGATGCTAAA |
| *CsAP2* | HD.10G0007680 | ACCAGGAAAGGAAGGCAAGT | CTCCAACGGGAAGTTGATGT |

**Supplementary Table 3** The change of EGCG, Theanine, Caffeine content in tea plants under different temperatures (%)

| **Temperature (℃)** | **15** | **20** | **25** | **30** |
| --- | --- | --- | --- | --- |
| EGCG | 4.27±0.11cC | 7.90±0.18bB | 9.46±0.07aA | 9.57±0.18aA |
| Theanine | 0.34±0.01cBC | 0.37±0.00aA | 0.35±0.01bB | 0.34±0.00cA |
| Caffeine | 1.29±0.04dD | 1.97±0.01cC | 2.76±0.02bB | 3.00±0.02aA |

**Note: Different lowercase letters represent *P*<0.05, which is significant between pairwise; Different capital letters represent *P*<0.01, which is extremely significant between pairwise.**

**Supplementary Table 4** The change of endogenous hormone content in tea plant under different temperature（ng/g)

| **Endogenous hormone** | **Temperature treatment (℃）** | | | |
| --- | --- | --- | --- | --- |
|  | **15** | **20** | **25** | **30** |
| IAA | 7.57±0.34dC | 8.68±0.17cB | 11.11±0.53bA | 12.03±0.21aA |
| IBA | 3.70±0.07aA | 3.70±0.14aA | 1.86±0.11bB | 0.78±0.03cC |
| IAA-Asp | 97.81±0.15bB | 97.45±0.14bB | 102.24±0.88aA | 28.39±0.19cC |
| auxins | 36.36±0.18bB | 36.61±0.14bB | 38.4±0.47aA | 13.73±0.13cC |
| GA1 | 0.94±0.04dD | 1.89±0.07bB | 1.60±0.09cC | 2.68±0.03aA |
| GA3 | 0.01±0.00cC | 0.08±0.01aA | 0.05±0.01bB | 0.07±0.00aA |
| GA4 | 1.04±0.02cC | 1.07±0.04cC | 1.32±0.03bB | 1.65±0.04aA |
| GAs | 1.99±0.06cC | 3.03±0.06bB | 2.98±0.09bB | 4.4±0.02aA |
| zeatin | 0.33±0.01aA | 0.35±0.03aA | 0.35±0.01aA | 0.32±0.01aA |
| tZR | 3.61±0.17dD | 4.41±0.07cC | 4.86±0.04bB | 6.95±0.18aA |
| iPR | 1.41±0.05aA | 0.57±0.02bB | 0.32±0.01cC | 0.23±0.02dD |
| iP | 0.22±0.00aA | 0.10±0.01cC | 0.09±0.01cC | 0.13±0.01bB |
| CTK | 5.57±0.17bB | 5.43±0.12bB | 5.43±0.12bB | 7.63±0.19aA |
| ABA | 465.64±12.86aA | 440.28±7.21bB | 305.33±7.57cC | 171.14±5.47dD |
| JA | 81.67±1.33cC | 240.16±3.00aA | 130.59±2.51bB | 26.67±0.87dD |
| JA-Ile | 3.54±0.32cC | 7.56±0.25aA | 4.49±0.27bB | 1.75±0.04dD |
| MeJA | 9.94±0.32bB | 23.20±0.38aA | 10.34±0.22bB | 1.21±0.06cC |
| JAs | 95.14±1.84cC | 270.91±3.37aA | 145.42±2.97bB | 29.64±0.85dD |
| SA | 850.66±43.03cC | 1765.37±98.51aA | 1549.37±14.02bB | 1548.65±56.21bB |
| MeSA | 0.30±0.02bB | 0.67±0.03aA | 0.30±0.01bB | 0.17±0.01cC |
| SAs | 850.96±43.04cC | 1766.05±98.51aA | 1549.67±14.03bB | 1548.82±56.21bB |

**Supplementary Table 5** The interpretation rate of the model for X and Y matrices

| **Model** | **Type** | **R2X(cum)** | **R2Y(cum)** | **Q2(cum)** |
| --- | --- | --- | --- | --- |
| 16 endogenous hormones and EGCG | OPLS | 0.963 | 0.989 | 0.971 |
| 16 endogenous hormones and Theanine | OPLS | 0.906 | 0.885 | 0.797 |
| 16 endogenous hormones and Caffeine | OPLS | 0.964 | 0.995 | 0.987 |

**Supplementary Table 6** Promoters prediction of 20 TFs and 7 structural genes of EGCG, theanine and caffeine biosynthesis

| **gene** | **element** | **sequence** | **cis-acting regulatory element** |
| --- | --- | --- | --- |
| *CsC2H2*(HD.04G0027010) | O2-site | GATGATGTGG | cis-acting regulatory element involved in zein metabolism regulation |
| *CsC2H2*(HD.04G0027010) | ABRE | TACGGTC | cis-acting element involved in the abscisic acid responsiveness |
| *CsC2H2*(HD.04G0027010) | GARE-motif | TCTGTTG | gibberellin-responsive element |
| *CsC2H2*(HD.04G0027010) | TGA-element | AACGAC | auxin-responsive element |
| *CsSBP*(HD.04G0020310) | O2-site | GTTGACGTGA | cis-acting regulatory element involved in zein metabolism regulation |
| *CsSBP*(HD.04G0020310) | TATC-box | TATCCCA | cis-acting element involved in gibberellin-responsiveness |
| *CsSBP*(HD.04G0020310) | TGACG-motif | TGACG | cis-acting regulatory element involved in the MeJA-responsiveness |
| *CsSBP*(HD.04G0020310) | TCA-element | CCATCTTTTT | cis-acting element involved in salicylic acid responsiveness |
| *CsSBP*(HD.04G0020310) | CGTCA-motif | CGTCA | cis-acting regulatory element involved in the MeJA-responsiveness |
| *CsSBP*(HD.04G0020310) | ABRE | ACGTG | cis-acting element involved in the abscisic acid responsiveness |
| *CsSBP*(HD.04G0020310) | ABRE | ACGTG | cis-acting element involved in the abscisic acid responsiveness |
| *CsERF*(HD.04G0001130) | O2-site | GATGATGTGG | cis-acting regulatory element involved in zein metabolism regulation |
| *CsERF*(HD.04G0001130) | O2-site | GATGATGTGG | cis-acting regulatory element involved in zein metabolism regulation |
| *CsERF*(HD.04G0001130) | TATC-box | TATCCCA | cis-acting element involved in gibberellin-responsiveness |
| *CsERF*(HD.04G0001130) | TGACG-motif | TGACG | cis-acting regulatory element involved in the MeJA-responsiveness |
| *CsERF*(HD.04G0001130) | TGACG-motif | TGACG | cis-acting regulatory element involved in the MeJA-responsiveness |
| *CsERF*(HD.04G0001130) | TCA-element | TCAGAAGAGG | cis-acting element involved in salicylic acid responsiveness |
| *CsERF*(HD.04G0001130) | ABRE | ACGTG | cis-acting element involved in the abscisic acid responsiveness |
| *CsERF*(HD.04G0001130) | CGTCA-motif | CGTCA | cis-acting regulatory element involved in the MeJA-responsiveness |
| *CsERF*(HD.04G0001130) | CGTCA-motif | CGTCA | cis-acting regulatory element involved in the MeJA-responsiveness |
| *CshD-ZIP*(HD.05G0010130) | TATC-box | TATCCCA | cis-acting element involved in gibberellin-responsiveness |
| *CshD-ZIP*(HD.05G0010130) | TGA-element | AACGAC | auxin-responsive element |
| *CshD-ZIP*(HD.05G0010130) | ABRE | CACGTG | cis-acting element involved in the abscisic acid responsiveness |
| *CshD-ZIP*(HD.05G0010130) | ABRE | ACGTG | cis-acting element involved in the abscisic acid responsiveness |
| *CsHD-ZIP*(HD.05G0006810) | TGACG-motif | TGACG | cis-acting regulatory element involved in the MeJA-responsiveness |
| *CsHD-ZIP*(HD.05G0006810) | TGACG-motif | TGACG | cis-acting regulatory element involved in the MeJA-responsiveness |
| *CsHD-ZIP*(HD.05G0006810) | TGA-element | AACGAC | auxin-responsive element |
| *CsHD-ZIP*(HD.05G0006810) | TGA-element | AACGAC | auxin-responsive element |
| *CsHD-ZIP*(HD.05G0006810) | CGTCA-motif | CGTCA | cis-acting regulatory element involved in the MeJA-responsiveness |
| *CsHD-ZIP*(HD.05G0006810) | CGTCA-motif | CGTCA | cis-acting regulatory element involved in the MeJA-responsiveness |
| *CsHD-ZIP*(HD.05G0006810) | ABRE | ACGTG | cis-acting element involved in the abscisic acid responsiveness |
| *CsHD-ZIP*(HD.05G0006810) | ABRE | CACGTG | cis-acting element involved in the abscisic acid responsiveness |
| *CsHD-ZIP*(HD.05G0006810) | ABRE | ACGTG | cis-acting element involved in the abscisic acid responsiveness |
| *CsHD-ZIP*(HD.05G0006810) | ABRE | GCAACGTGTC | cis-acting element involved in the abscisic acid responsiveness |
| *CsbZIP*(HD.05G0001910) | O2-site | GATGA(C/T)(A/G)TG(A/G) | cis-acting regulatory element involved in zein metabolism regulation |
| *CsbZIP*(HD.05G0001910) | TCA-element | TCAGAAGAGG | cis-acting element involved in salicylic acid responsiveness |
| *CsERF*(HD.06G0019520) | CGTCA-motif | CGTCA | cis-acting regulatory element involved in the MeJA-responsiveness |
| *CsERF*(HD.06G0019520) | CGTCA-motif | CGTCA | cis-acting regulatory element involved in the MeJA-responsiveness |
| *CsERF*(HD.06G0019520) | CGTCA-motif | CGTCA | cis-acting regulatory element involved in the MeJA-responsiveness |
| *CsERF*(HD.06G0019520) | CGTCA-motif | CGTCA | cis-acting regulatory element involved in the MeJA-responsiveness |
| *CsERF*(HD.06G0019520) | CGTCA-motif | CGTCA | cis-acting regulatory element involved in the MeJA-responsiveness |
| *CsERF*(HD.06G0019520) | ABRE | ACGTG | cis-acting element involved in the abscisic acid responsiveness |
| *CsERF*(HD.06G0019520) | ABRE | CACGTG | cis-acting element involved in the abscisic acid responsiveness |
| *CsERF*(HD.06G0019520) | ABRE | ACGTG | cis-acting element involved in the abscisic acid responsiveness |
| *CsERF*(HD.06G0019520) | TCA-element | CCATCTTTTT | cis-acting element involved in salicylic acid responsiveness |
| *CsERF*(HD.06G0019520) | TGACG-motif | TGACG | cis-acting regulatory element involved in the MeJA-responsiveness |
| *CsERF*(HD.06G0019520) | TGACG-motif | TGACG | cis-acting regulatory element involved in the MeJA-responsiveness |
| *CsERF*(HD.06G0019520) | TGACG-motif | TGACG | cis-acting regulatory element involved in the MeJA-responsiveness |
| *CsERF*(HD.06G0019520) | TGACG-motif | TGACG | cis-acting regulatory element involved in the MeJA-responsiveness |
| *CsERF*(HD.06G0019520) | TGACG-motif | TGACG | cis-acting regulatory element involved in the MeJA-responsiveness |
| *CsERF*(HD.06G0019520) | GARE-motif | TCTGTTG | gibberellin-responsive element |
| *CsERF*(HD.06G0016380) | TATC-box | TATCCCA | cis-acting element involved in gibberellin-responsiveness |
| *CsERF*(HD.06G0016380) | TGACG-motif | TGACG | cis-acting regulatory element involved in the MeJA-responsiveness |
| *CsERF*(HD.06G0016380) | TGACG-motif | TGACG | cis-acting regulatory element involved in the MeJA-responsiveness |
| *CsERF*(HD.06G0016380) | TGACG-motif | TGACG | cis-acting regulatory element involved in the MeJA-responsiveness |
| *CsERF*(HD.06G0016380) | CGTCA-motif | CGTCA | cis-acting regulatory element involved in the MeJA-responsiveness |
| *CsERF*(HD.06G0016380) | CGTCA-motif | CGTCA | cis-acting regulatory element involved in the MeJA-responsiveness |
| *CsERF*(HD.06G0016380) | CGTCA-motif | CGTCA | cis-acting regulatory element involved in the MeJA-responsiveness |
| *CsERF*(HD.06G0016380) | ABRE | ACGTG | cis-acting element involved in the abscisic acid responsiveness |
| *CsERF*(HD.06G0016380) | ABRE | CACGTG | cis-acting element involved in the abscisic acid responsiveness |
| *CsERF*(HD.06G0016380) | ABRE | ACGTG | cis-acting element involved in the abscisic acid responsiveness |
| *CsERF*(HD.06G0016380) | ABRE | ACGTG | cis-acting element involved in the abscisic acid responsiveness |
| *CsERF*(HD.06G0016380) | ABRE | CACGTG | cis-acting element involved in the abscisic acid responsiveness |
| *CsERF*(HD.06G0016380) | ABRE | ACGTG | cis-acting element involved in the abscisic acid responsiveness |
| *CsERF*(HD.06G0016380) | ABRE | ACGTG | cis-acting element involved in the abscisic acid responsiveness |
| *CsERF*(HD.06G0016330) | GARE-motif | TCTGTTG | gibberellin-responsive element |
| *CsERF*(HD.06G0016330) | GARE-motif | TCTGTTG | gibberellin-responsive element |
| *CsERF*(HD.06G0016330) | GARE-motif | TCTGTTG | gibberellin-responsive element |
| *CsERF*(HD.06G0016330) | TCA-element | CCATCTTTTT | cis-acting element involved in salicylic acid responsiveness |
| *CsERF*(HD.06G0016330) | ABRE | ACGTG | cis-acting element involved in the abscisic acid responsiveness |
| *CsAP2*(HD.06G0038000) | TATC-box | TATCCCA | cis-acting element involved in gibberellin-responsiveness |
| *CsAP2*(HD.06G0038000) | CGTCA-motif | CGTCA | cis-acting regulatory element involved in the MeJA-responsiveness |
| *CsAP2*(HD.06G0038000) | ABRE | ACGTG | cis-acting element involved in the abscisic acid responsiveness |
| *CsAP2*(HD.06G0038000) | ABRE | ACGTG | cis-acting element involved in the abscisic acid responsiveness |
| *CsAP2*(HD.06G0038000) | TGACG-motif | TGACG | cis-acting regulatory element involved in the MeJA-responsiveness |
| *CsERF*(HD.02G0007290) | TCA-element | CCATCTTTTT | cis-acting element involved in salicylic acid responsiveness |
| *CsERF*(HD.02G0007290) | TGACG-motif | TGACG | cis-acting regulatory element involved in the MeJA-responsiveness |
| *CsERF*(HD.02G0007290) | TGACG-motif | TGACG | cis-acting regulatory element involved in the MeJA-responsiveness |
| *CsERF*(HD.02G0007290) | CGTCA-motif | CGTCA | cis-acting regulatory element involved in the MeJA-responsiveness |
| *CsERF*(HD.02G0007290) | CGTCA-motif | CGTCA | cis-acting regulatory element involved in the MeJA-responsiveness |
| *CsERF*(HD.02G0007290) | O2-site | GATGATGTGG | cis-acting regulatory element involved in zein metabolism regulation |
| *CsC2H2*(HD.02G0015370) | TCA-element | TCAGAAGAGG | cis-acting element involved in salicylic acid responsiveness |
| *CsC2H2*(HD.02G0015370) | TGACG-motif | TGACG | cis-acting regulatory element involved in the MeJA-responsiveness |
| *CsC2H2*(HD.02G0015370) | CGTCA-motif | CGTCA | cis-acting regulatory element involved in the MeJA-responsiveness |
| *CsC2H2*(HD.02G0015370) | AuxRR-core | GGTCCAT | cis-acting regulatory element involved in auxin responsiveness |
| *CsC2H2*(HD.02G0015370) | TATC-box | TATCCCA | cis-acting element involved in gibberellin-responsiveness |
| *CsWRKY*(HD.08002776) | AuxRR-core | GGTCCAT | cis-acting regulatory element involved in auxin responsiveness |
| *CsWRKY*(HD.08002776) | TGACG-motif | TGACG | cis-acting regulatory element involved in the MeJA-responsiveness |
| *CsWRKY*(HD.08002776) | TGACG-motif | TGACG | cis-acting regulatory element involved in the MeJA-responsiveness |
| *CsWRKY*(HD.08002776) | TGACG-motif | TGACG | cis-acting regulatory element involved in the MeJA-responsiveness |
| *CsWRKY*(HD.08002776) | TGACG-motif | TGACG | cis-acting regulatory element involved in the MeJA-responsiveness |
| *CsWRKY*(HD.08002776) | TGACG-motif | TGACG | cis-acting regulatory element involved in the MeJA-responsiveness |
| *CsWRKY*(HD.08002776) | ABRE | ACGTG | cis-acting element involved in the abscisic acid responsiveness |
| *CsWRKY*(HD.08002776) | ABRE | TACGTGTC | cis-acting element involved in the abscisic acid responsiveness |
| *CsWRKY*(HD.08002776) | ABRE | ACGTG | cis-acting element involved in the abscisic acid responsiveness |
| *CsWRKY*(HD.08002776) | CGTCA-motif | CGTCA | cis-acting regulatory element involved in the MeJA-responsiveness |
| *CsWRKY*(HD.08002776) | CGTCA-motif | CGTCA | cis-acting regulatory element involved in the MeJA-responsiveness |
| *CsWRKY*(HD.08002776) | CGTCA-motif | CGTCA | cis-acting regulatory element involved in the MeJA-responsiveness |
| *CsWRKY*(HD.08002776) | CGTCA-motif | CGTCA | cis-acting regulatory element involved in the MeJA-responsiveness |
| *CsWRKY*(HD.08002776) | CGTCA-motif | CGTCA | cis-acting regulatory element involved in the MeJA-responsiveness |
| *CsAP2*(HD.10G0007680) | TGACG-motif | TGACG | cis-acting regulatory element involved in the MeJA-responsiveness |
| *CsAP2*(HD.10G0007680) | ABRE | ACGTG | cis-acting element involved in the abscisic acid responsiveness |
| *CsAP2*(HD.10G0007680) | ABRE | ACGTG | cis-acting element involved in the abscisic acid responsiveness |
| *CsAP2*(HD.10G0007680) | CGTCA-motif | CGTCA | cis-acting regulatory element involved in the MeJA-responsiveness |
| *CsAP2*(HD.10G0007680) | SARE | TTCGACCATCTT | cis-acting element involved in salicylic acid responsiveness |
| *CsGRF*(HD.11G0012770) | TATC-box | TATCCCA | cis-acting element involved in gibberellin-responsiveness |
| *CsGRF*(HD.11G0012770) | ABRE | ACGTG | cis-acting element involved in the abscisic acid responsiveness |
| *CsGRF*(HD.11G0012770) | TCA-element | CCATCTTTTT | cis-acting element involved in salicylic acid responsiveness |
| *CsWRKY*(HD.09G0023240) | O2-site | GATGATGTGG | cis-acting regulatory element involved in zein metabolism regulation |
| *CsWRKY*(HD.09G0023240) | TATC-box | TATCCCA | cis-acting element involved in gibberellin-responsiveness |
| *CsWRKY*(HD.09G0023240) | TGA-element | AACGAC | auxin-responsive element |
| *CsWRKY*(HD.09G0023240) | ABRE | ACGTG | cis-acting element involved in the abscisic acid responsiveness |
| *CsMYB_related*(HD.15G0003730) | TATC-box | TATCCCA | cis-acting element involved in gibberellin-responsiveness |
| *CsMYB_related*(HD.15G0003730) | CGTCA-motif | CGTCA | cis-acting regulatory element involved in the MeJA-responsiveness |
| *CsMYB_related*(HD.15G0003730) | CGTCA-motif | CGTCA | cis-acting regulatory element involved in the MeJA-responsiveness |
| *CsMYB_related*(HD.15G0003730) | ABRE | GACACGTGGC | cis-acting element involved in the abscisic acid responsiveness |
| *CsMYB_related*(HD.15G0003730) | ABRE | CACGTG | cis-acting element involved in the abscisic acid responsiveness |
| *CsMYB_related*(HD.15G0003730) | ABRE | ACGTG | cis-acting element involved in the abscisic acid responsiveness |
| *CsMYB_related*(HD.15G0003730) | TGA-element | AACGAC | auxin-responsive element |
| *CsMYB_related*(HD.15G0003730) | TGA-element | AACGAC | auxin-responsive element |
| *CsMYB_related*(HD.15G0003730) | TGACG-motif | TGACG | cis-acting regulatory element involved in the MeJA-responsiveness |
| *CsMYB_related*(HD.15G0003730) | TGACG-motif | TGACG | cis-acting regulatory element involved in the MeJA-responsiveness |
| *CsERF*(HD.01G0012310) | P-box | CCTTTTG | gibberellin-responsive element |
| *CsERF*(HD.01G0012310) | CGTCA-motif | CGTCA | cis-acting regulatory element involved in the MeJA-responsiveness |
| *CsERF*(HD.01G0012310) | CGTCA-motif | CGTCA | cis-acting regulatory element involved in the MeJA-responsiveness |
| *CsERF*(HD.01G0012310) | CGTCA-motif | CGTCA | cis-acting regulatory element involved in the MeJA-responsiveness |
| *CsERF*(HD.01G0012310) | CGTCA-motif | CGTCA | cis-acting regulatory element involved in the MeJA-responsiveness |
| *CsERF*(HD.01G0012310) | TGACG-motif | TGACG | cis-acting regulatory element involved in the MeJA-responsiveness |
| *CsERF*(HD.01G0012310) | TGACG-motif | TGACG | cis-acting regulatory element involved in the MeJA-responsiveness |
| *CsERF*(HD.01G0012310) | TGACG-motif | TGACG | cis-acting regulatory element involved in the MeJA-responsiveness |
| *CsERF*(HD.01G0012310) | TGACG-motif | TGACG | cis-acting regulatory element involved in the MeJA-responsiveness |
| *CsERF*(HD.01G0012310) | ABRE | AACCCGG | cis-acting element involved in the abscisic acid responsiveness |
| *CsERF*(HD.01G0012310) | ABRE | ACGTG | cis-acting element involved in the abscisic acid responsiveness |
| *CsERF*(HD.01G0012310) | ABRE | CACGTG | cis-acting element involved in the abscisic acid responsiveness |
| *CsERF*(HD.01G0012310) | ABRE | ACGTG | cis-acting element involved in the abscisic acid responsiveness |
| *CsWRKY*(HD.07G0017570) | P-box | CCTTTTG | gibberellin-responsive element |
| *CsWRKY*(HD.07G0017570) | CGTCA-motif | CGTCA | cis-acting regulatory element involved in the MeJA-responsiveness |
| *CsWRKY*(HD.07G0017570) | CGTCA-motif | CGTCA | cis-acting regulatory element involved in the MeJA-responsiveness |
| *CsWRKY*(HD.07G0017570) | CGTCA-motif | CGTCA | cis-acting regulatory element involved in the MeJA-responsiveness |
| *CsWRKY*(HD.07G0017570) | CGTCA-motif | CGTCA | cis-acting regulatory element involved in the MeJA-responsiveness |
| *CsWRKY*(HD.07G0017570) | TCA-element | TCAGAAGAGG | cis-acting element involved in salicylic acid responsiveness |
| *CsWRKY*(HD.07G0017570) | TCA-element | CCATCTTTTT | cis-acting element involved in salicylic acid responsiveness |
| *CsWRKY*(HD.07G0017570) | O2-site | GTTGACGTGA | cis-acting regulatory element involved in zein metabolism regulation |
| *CsWRKY*(HD.07G0017570) | TGACG-motif | TGACG | cis-acting regulatory element involved in the MeJA-responsiveness |
| *CsWRKY*(HD.07G0017570) | TGACG-motif | TGACG | cis-acting regulatory element involved in the MeJA-responsiveness |
| *CsWRKY*(HD.07G0017570) | TGACG-motif | TGACG | cis-acting regulatory element involved in the MeJA-responsiveness |
| *CsWRKY*(HD.07G0017570) | TGACG-motif | TGACG | cis-acting regulatory element involved in the MeJA-responsiveness |
| *CsWRKY*(HD.07G0017570) | ABRE | ACGTG | cis-acting element involved in the abscisic acid responsiveness |
| *CsWRKY*(HD.07G0017570) | ABRE | ACGTG | cis-acting element involved in the abscisic acid responsiveness |
| *CsWRKY*(HD.07G0017570) | ABRE | ACGTG | cis-acting element involved in the abscisic acid responsiveness |
| *CsANS*(HD.09G0021220) | TCA-element | TCAGAAGAGG | cis-acting element involved in salicylic acid responsiveness |
| *CsANS*(HD.09G0021220) | ABRE | CACGTG | cis-acting element involved in the abscisic acid responsiveness |
| *CsANS*(HD.09G0021220) | ABRE | ACGTG | cis-acting element involved in the abscisic acid responsiveness |
| *CsANS*(HD.12G0014070) | TGACG-motif | TGACG | cis-acting regulatory element involved in the MeJA-responsiveness |
| *CsANS*(HD.12G0014070) | CGTCA-motif | CGTCA | cis-acting regulatory element involved in the MeJA-responsiveness |
| *CsANS*(HD.12G0014070) | ABRE | CACGTG | cis-acting element involved in the abscisic acid responsiveness |
| *CsANS*(HD.12G0014070) | ABRE | ACGTG | cis-acting element involved in the abscisic acid responsiveness |
| *CsANS*(HD.12G0014070) | ABRE | ACGTG | cis-acting element involved in the abscisic acid responsiveness |
| *CsANS*(HD.12G0014070) | ABRE | CACGTG | cis-acting element involved in the abscisic acid responsiveness |
| *CsANS*(HD.12G0014070) | ABRE | ACGTG | cis-acting element involved in the abscisic acid responsiveness |
| *CsANS*(HD.12G0014070) | O2-site | GATGA(C/T)(A/G)TG(A/G) | cis-acting regulatory element involved in zein metabolism regulation |
| *CsC4H*(HD.14G0014170) | GARE-motif | TCTGTTG | gibberellin-responsive element |
| *CsC4H*(HD.14G0014170) | O2-site | GATGATGTGG | cis-acting regulatory element involved in zein metabolism regulation |
| *CsC4H*(HD.14G0014170) | O2-site | GATGA(C/T)(A/G)TG(A/G) | cis-acting regulatory element involved in zein metabolism regulation |
| *CsC4H*(HD.14G0014170) | TGA-element | AACGAC | auxin-responsive element |
| *CsCHS*(HD.10G0022640) | AuxRR-core | GGTCCAT | cis-acting regulatory element involved in auxin responsiveness |
| *CsCHS*(HD.10G0022640) | ABRE | CGCACGTGTC | cis-acting element involved in the abscisic acid responsiveness |
| *CsCHS*(HD.10G0022640) | ABRE | CACGTG | cis-acting element involved in the abscisic acid responsiveness |
| *CsCHS*(HD.10G0022640) | ABRE | ACGTG | cis-acting element involved in the abscisic acid responsiveness |
| *CsCHS*(HD.10G0022640) | ABRE | CACGTG | cis-acting element involved in the abscisic acid responsiveness |
| *CsCHS*(HD.10G0022640) | ABRE | ACGTG | cis-acting element involved in the abscisic acid responsiveness |
| *CsCHS*(HD.10G0022640) | ABRE | ACGTG | cis-acting element involved in the abscisic acid responsiveness |
| *CsCHS*(HD.10G0022640) | TCA-element | CCATCTTTTT | cis-acting element involved in salicylic acid responsiveness |
| *CsCA*(HD.06G0022520) | TATC-box | TATCCCA | cis-acting element involved in gibberellin-responsiveness |
| *CsCA*(HD.06G0022520) | TGACG-motif | TGACG | cis-acting regulatory element involved in the MeJA-responsiveness |
| *CsCA*(HD.06G0022520) | TGACG-motif | TGACG | cis-acting regulatory element involved in the MeJA-responsiveness |
| *CsCA*(HD.06G0022520) | TGACG-motif | TGACG | cis-acting regulatory element involved in the MeJA-responsiveness |
| *CsCA*(HD.06G0022520) | TCA-element | CCATCTTTTT | cis-acting element involved in salicylic acid responsiveness |
| *CsCA*(HD.06G0022520) | TCA-element | CCATCTTTTT | cis-acting element involved in salicylic acid responsiveness |
| *CsCA*(HD.06G0022520) | CGTCA-motif | CGTCA | cis-acting regulatory element involved in the MeJA-responsiveness |
| *CsCA*(HD.06G0022520) | CGTCA-motif | CGTCA | cis-acting regulatory element involved in the MeJA-responsiveness |
| *CsCA*(HD.06G0022520) | CGTCA-motif | CGTCA | cis-acting regulatory element involved in the MeJA-responsiveness |
| *CsCA*(HD.06G0022520) | ABRE | ACGTG | cis-acting element involved in the abscisic acid responsiveness |
| *CsCA*(HD.06G0022520) | ABRE | ACGTG | cis-acting element involved in the abscisic acid responsiveness |
| *CsCA*(HD.06G0022520) | ABRE | ACGTG | cis-acting element involved in the abscisic acid responsiveness |
| *CsgadB*(HD.01G0021590) | CGTCA-motif | CGTCA | cis-acting regulatory element involved in the MeJA-responsiveness |
| *CsgadB*(HD.01G0021590) | TGA-element | AACGAC | auxin-responsive element |
| *CsgadB*(HD.01G0021590) | TGACG-motif | TGACG | cis-acting regulatory element involved in the MeJA-responsiveness |
| *CsgadB*(HD.01G0021590) | ABRE | ACGTG | cis-acting element involved in the abscisic acid responsiveness |
| *CsgadB*(HD.01G0021590) | ABRE | ACGTG | cis-acting element involved in the abscisic acid responsiveness |
| *CsgadB*(HD.01G0021590) | ABRE | ACGTG | cis-acting element involved in the abscisic acid responsiveness |
| *CsgadB*(HD.01G0021590) | ABRE | ACGTG | cis-acting element involved in the abscisic acid responsiveness |
| *CsgadB*(HD.01G0021590) | ABRE | ACGTG | cis-acting element involved in the abscisic acid responsiveness |
| *CsgadB*(HD.01G0021590) | CGTCA-motif | CGTCA | cis-acting regulatory element involved in the MeJA-responsiveness |
| *CsgadB*(HD.01G0021590) | TGA-element | AACGAC | auxin-responsive element |
| *CsgadB*(HD.01G0021590) | TGACG-motif | TGACG | cis-acting regulatory element involved in the MeJA-responsiveness |
| *CsgadB*(HD.01G0021590) | ABRE | ACGTG | cis-acting element involved in the abscisic acid responsiveness |
| *CsgadB*(HD.01G0021590) | ABRE | ACGTG | cis-acting element involved in the abscisic acid responsiveness |
| *CsgadB*(HD.01G0021590) | ABRE | ACGTG | cis-acting element involved in the abscisic acid responsiveness |
| *CsgadB*(HD.01G0021590) | ABRE | ACGTG | cis-acting element involved in the abscisic acid responsiveness |
| *CsgadB*(HD.01G0021590) | ABRE | ACGTG | cis-acting element involved in the abscisic acid responsiveness |
| *CspurA*(HD.11G0025240) | ABRE | ACGTG | cis-acting element involved in the abscisic acid responsiveness |
| *CspurA*(HD.11G0025240) | CGTCA-motif | CGTCA | cis-acting regulatory element involved in the MeJA-responsiveness |
| *CspurA*(HD.11G0025240) | CGTCA-motif | CGTCA | cis-acting regulatory element involved in the MeJA-responsiveness |
| *CspurA*(HD.11G0025240) | CGTCA-motif | CGTCA | cis-acting regulatory element involved in the MeJA-responsiveness |
| *CspurA*(HD.11G0025240) | CGTCA-motif | CGTCA | cis-acting regulatory element involved in the MeJA-responsiveness |
| *CspurA*(HD.11G0025240) | TGACG-motif | TGACG | cis-acting regulatory element involved in the MeJA-responsiveness |
| *CspurA*(HD.11G0025240) | TGACG-motif | TGACG | cis-acting regulatory element involved in the MeJA-responsiveness |
| *CspurA*(HD.11G0025240) | TGACG-motif | TGACG | cis-acting regulatory element involved in the MeJA-responsiveness |
| *CspurA*(HD.11G0025240) | TGACG-motif | TGACG | cis-acting regulatory element involved in the MeJA-responsiveness |

# Supplementary Figure


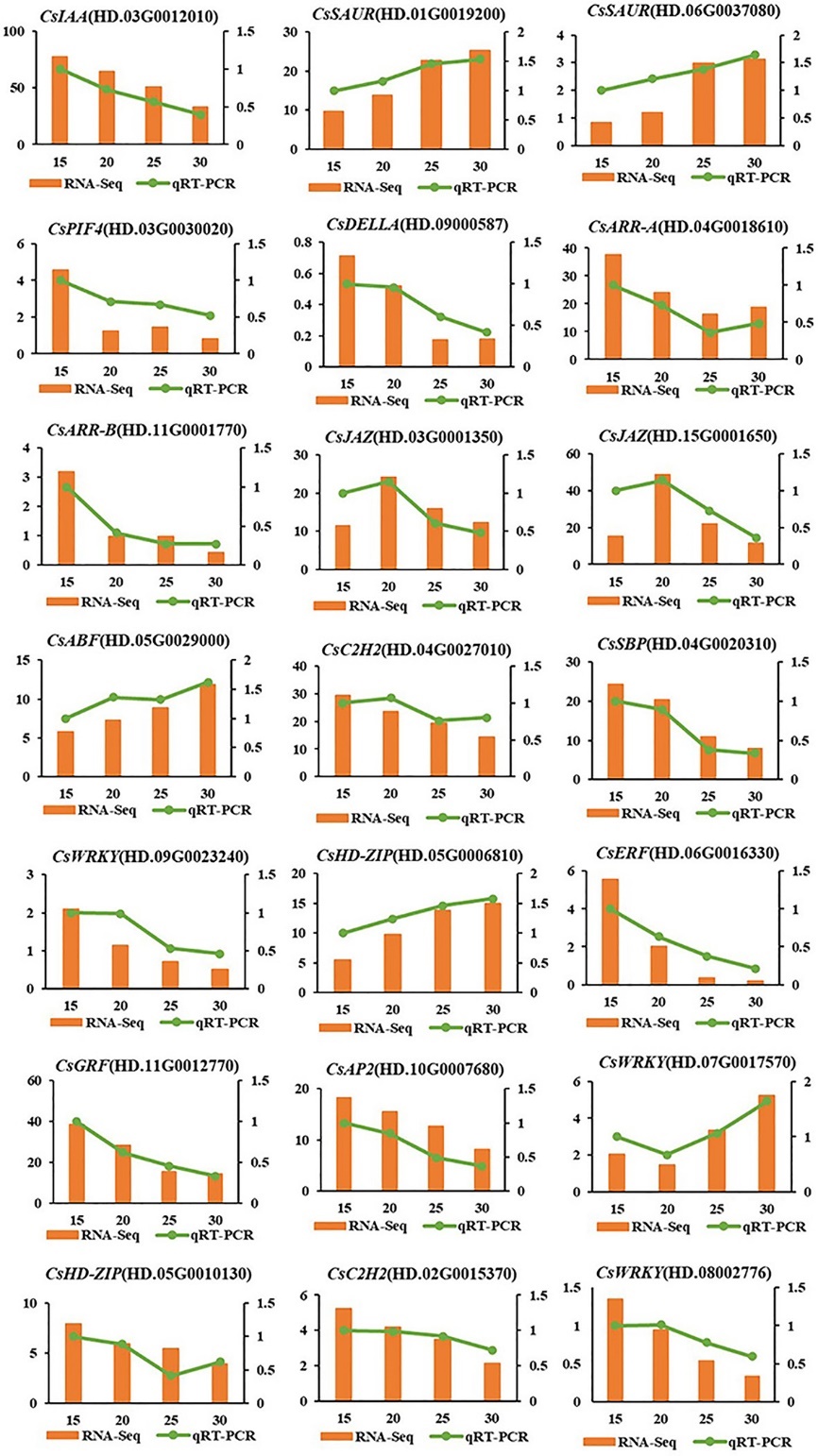


**Supplementary Figure 1** The expression of remaining 21 core genes by RNA-seq and qRT-PCR.
